# *Pseudomonas forestsoilum* sp. nov. and *P. tohonis* biocontrol bacterial wilt by quenching 3-hydroxypalmitic acid methyl ester

**DOI:** 10.3389/fpls.2023.1193297

**Published:** 2023-06-30

**Authors:** Si Wang, Ming Hu, Huilin Chen, Chuhao Li, Yang Xue, Xinyue Song, Yuqing Qi, Fan Liu, Xiaofan Zhou, Lian-hui Zhang, Jianuan Zhou

**Affiliations:** National Key Laboratory of Green Pesticide, Guangdong Province Key Laboratory of Microbial Signals and Disease Control, Integrative Microbiology Research Center, South China Agricultural University, Guangzhou, China

**Keywords:** 3-hydroxypalmitic acid methyl ester, quorum quenching, *Ralstonia solanacearum*, bacterial wilt, *Pseudomonas*

## Abstract

Bacterial wilt caused by *Ralstonia solanacearum* ranks the second top important bacterial plant disease worldwide. It is also the most important bacterial disease threatening the healthy development of *Casuarina equisetifolia* protection forest. 3-hydroxypalmitic acid methyl ester (3-OH PAME) functions as an important quorum sensing (QS) signal regulating the expression of virulence genes in *R. solanacearum*, and has been regarded as an ideal target for disease prevention and control. To screen native microorganisms capable of degrading 3-OH PAME, samples of *C. equisetifolia* branches and forest soil were collected and cultured in the medium containing 3-OH PAME as the sole carbon source. Bacteria with over 85% degradation rates of 3-OH PAME after 7-day incubation were further separated and purified. As a result, strain Q1-7 isolated from forest soil and strain Q4-3 isolated from *C. equisetifolia* branches were obtained and identified as *Pseudomonas* novel species *Pseudomonas forestsoilum* sp. nov. and *P. tohonis*, respectively, according to whole genome sequencing results. The degradation efficiencies of 3-OH PAME of strains Q1-7 and Q4-3 were 95.80% and 100.00% at 48 h, respectively. Both strains showed high esterase activities and inhibited *R. solanacearum* exopolysaccharide (EPS) and cellulase production. Application of strains Q1-7 and Q4-3 effectively protects *C. equisetifolia*, peanut and tomato plants from infection by *R. solanacearum*. Findings in this study provide potential resources for the prevention and control of bacterial wilt caused by *R. solanacearum*, as well as valuable materials for the identification of downstream quenching genes and the research and development of quenching enzymes for disease control.

## Introduction

*Ralstonia solanacearum* is one of the top ten important plant pathogenic bacteria in the world (Mansfield et al., 2012). It infects a wild range of host plants with more than 450 species in 54 families (Wicker et al., 2007), including crops of potato, cassava, soybean, pumpkin, tomato, pepper, eggplant, peanut, tobacco, strawberry, banana, ginger etc., and woody plants such as eucalyptus, mulberry, olive and *Casuarina equisetifolia* etc. (Hayward, 1991). The bacterial wilt incidence of eucalyptus ranges from 20% to 40% in Guangdong and Hainan to 90% in Fujian (Wu et al., 2007), which in *Casuarina*, varies around 50% to 90% in China (Sun et al., 2013). Different strains isolated from different regions and hosts exhibit rich intraspecific genetic diversity in terms of host range and pathogenicity (Hayward, 1991; Peeters et al., 2013; Huet, 2014; Li et al., 2016), increasing the difficulty and complexity for pathogen pathogenicity research and disease control.

Virulence of *R. solanacearum* is determined mainly by extracellular polysaccharides (EPS) and proteases, motility and chemotaxis driven by flagella, pili and fimbria, and effectors translocated by secretion systems (Huang and Allen, 1997; Tans-Kersten et al., 2001; Yao and Allen, 2007; Milling et al., 2011; Marchetti et al., 2017). Once invading the damaged or natural openings at the plant root margin, *R. solanacearum* cells first attach to the tissue surface. During this early stage of infestation, the pathogen senses plant signals through the outer membrane receptor protein PrhA, and sequentially transmits and activates the regulatory genes *prhJ* and *hrpG*, and eventually initiates the expression of T3SS and its effectors (Genin and Denny, 2012). Meanwhile, a LysR family transcriptional regulator PhcA represses the expression of *prhR* and *pehSR*, cell motility and the production of PehA and siderophore. After avoiding the plant’s innate immune response (Nakano et al., 2013; Hikichi et al., 2017; Mori et al., 2018b), the cells rapidly multiply and activate the population sensing system to produce mushroom-like biofilms essential for the adhesion and virulence of *R. solanacearum* (Mori et al., 2016), from which planktonic bacterial cells are released into the xylem ducts (Mori et al., 2016; Hikichi et al., 2017; Mori et al., 2018a), and then grow rapidly and expand in the xylem to activate *phc* system to synthesize 3-hydroxypalmitic acid methyl ester (3-OH PAME) or (*R*)-methyl 3-hydroxymyristate [(*R*)-3-OH MAME] quorum sensing (QS) signal (Flavier et al., 1997; Kai et al., 2015). Once the concentration of the QS signal reaches a threshold (5 nM), it is sensed by the histidine kinase PhcS/PhcB that activates PhcR by phosphorylation (Clough et al., 1997b), which then releases the post-transcriptional repression of PhcA. The increased activity of PhcA turns on the expression of EPS via XpsR, cellulase encoding genes *egl* and *cbhA*, pectin esterase encoding gene *pme*, as well as another set of QS system mediated by AHL (Genin and Denny, 2012) and induce plant pathogenesis (Vesse et al., 1995; Genin and Denny, 2012; Hikichi et al., 2017). The AHL signal is synthesized by the SolI and bound to the SolR transcriptional regulator once it reaches a threshold (Flavier et al., 1997). Interestingly, inactivation of *solI*/*solR* in *R. solanacearum* did not alter bacterial pathogenicity and the virulence factors produced, whereas, mutation of *phcA* resulted in the loss of pathogenicity and the dramatically reduced production of EPS and proteases (Brumbley et al., 1993; Perrier et al., 2018).

Since PhcA is located at the core of the regulation network of *R. solanacearum*, and its activity is regulated by 3-OH-PAME molecule, quenching of 3-OH-PAME or (*R*)-3-OH MAME is one of the effective ways to control bacterial wilt caused by *R. solanacearum*. Previous study isolated *Ideonella* sp. strain 0-0013 from soil using selective-enrichment culture method as a quorum quenching (QQ) agent that produces *β*-hydroxypalmitate methyl ester hydrolase (*β*HPMEH) to degrade 3-OH-PAME, thereby suppressing the production of EPS and virulence of *R. solanacearum* (Shinohara et al., 2007). A recent study identified novel esterases/lipases using soil metagenome sequencing that display various levels of hydrolytic activities towards 3-OH-PAME, and decrease the EPS production without affecting bacterial growth (Lee et al., 2018).

*Casuarina equisetifolia* is an important coastal protection woody plant in southeast China. In 1960s and 1970s, bacterial wilt caused by *R. solanacearum* resulted in a massive die-off of *Casuarina* in the coastal regions of Guangdong province. The disease was later controlled by planting *R. solanacearum*-resistant asexual *Casuarina* line 701 (Guo and Liang, 1986; Liang, 1986). However, in recent years, resurgence of *Casuarina* bacterial wilt emergences in Wuchuan, Yangjiang, Maoming, Zhanjiang, Jieyang and Shantou cities in Guangdong province, and Xinhai Forest in Hainan province (Liu, 2016). In Wuchuan city, it resulted in a devastating death of more than 7,000 acres of *Casuarina* forest (Liu, 2016). The later cultivated A13 and A8 asexual lines have also gradually lost their resistance to *R. solanacearum* in the field (Xu et al., 2017). The resurgence of *Casuarina* bacterial wilt suggests that the plant resistance has been overcome through long-term environmental adaptation and genomic mutation of *R. solanacearum*. In this study, in order to develop biological control measures against *Casuarina* bacterial wilt, we used 3-OH PAME molecule as the sole carbon source for enrichment culturing the native microorganisms obtained from *C. equisetifolia* planting forest in Haitouwan Forest in Zhanjiang city, Guangdong province. Two *Pseudomonas* strains Q1-7 and Q4-3 showed great degradative activities towards 3-OH PAME and eliminated the *phc* system regulated traits, and performed great control efficiency against bacterial wilts of plant hosts caused by *R. solanacearum*.

## Materials and methods

### Chemicals and plants

The pure 3-OH PAME chemical was synthesized by Shanghai Zixia Biotechnology Co., Ltd. and the chromatographic methanol was used to dissolve it. All other chemicals were synthesized by Guangzhou Dingguo Biotechnology Co. LTD. The plants used in the following inoculation experiments (*Casuarina equisetifolia* cv. Wenchang, tomato cv. Provence, and peanut cv. Luhua) were purchased or self-cultivated.

### Strains cultivation conditions and medium preparation

The phytopathogenic bacterium used in this study was *R. solanacearum* NS25, which was isolated from *Casuarina* bacterial wilting trunk previously, and cultured in CPG medium (peptone, 10.0 g/L; glucose, 5.0 g/L; and casamino acid, 1.0 g/L; solid medium added with 15 g/L agar; Ph 7.0) at 30°C (Hendrick and Sequeira, 1984). The TTC medium was added with 5% 2,3,5-triphenyltetrazolium chloride (TTC) in a 1:1000 ratio to the melted CPG solid medium. The QQ strains Q1-7 and Q4-3 were cultivated in LB medium (NaCl, 10.0 g/L; tryptone, 10.0 g/L; and yeast extract, 5.0 g/L; solid medium added with 15 g/L agar; Ph 7.0) at 28°C (Maniatis et al., 1982).

The mineral salt medium [MSM; (NH_4_)_2_SO_4_, 2.0 g/L; Na_2_HPO_4_·12H_2_O, 1.5 g/L; NaH_2_PO_4_, 1.5 g/L; MgSO_4_·7H_2_O, 0.2 g/L; CaCl_2_·2H_2_O, 0.01 g/L; FeSO_4_·7H_2_O, 0.001 g/L; Ph 6.5] was used in the QQ bacterial screening and degradation tests of 3-OH PAME (Zhang et al., 2021).

The medium for swimming motility test contains 5.0 g/L bactopeptone, 5.0 g/L NaCl, and 3 g/L agar, that for swarming test contains 0.5% M8 plates supplemented with 2.0 g/L glucose and 0.5 g/L glutamate, and that for twitching test contains 10.0 g/L NaCl, 10.0 g/L tryptone, 5.0 g/L yeast extract and 15 g/L agar (Murray and Kazmierczak, 2006; Hu et al., 2021). SOBG medium for the measurement of biofilm formation contains 20.0 g/L tryptone, 5.0 g/L yeast extract, 2.4 g/L MgSO_4_·7H_2_O, 0.5 g/L NaCl, 0.186 g/L KCl, and 2% glycerol (Branda et al., 2005; Hu et al., 2021).

The medium for the measurement of EPS production (YEB) contains 10.0 g/L tryptone, 5.0 g/L yeast extract, 5 g/L KCl, 0.5 g/L MgSO_4_·7H_2_O, 5 g/L sucrose, 15 g/L agar (Romling et al., 1998). The medium for the measurement of pectate lyase (Pel) activity contains 10.0 g/L polygalacturonic acid, 10.0 g/L yeast extract, 8.0 g/L agar, 4.8448 g/L Tris-HCl, and 0.1125 g/L CaCl_2_. The medium for the measurement of polygalacturonase (Peh) activity contains 5.0 g/L polygalacturonic acid, 2.0 g/L sucrose, 15.0 g/L agar, and 2.0 g/L (NH_4_)_2_SO_4_. The medium for the measurement of cellulase (Cel) activity contains 1.0 g/L carboxymethyl cellulose, 3.8 g/L Na_3_PO_4_, and 8.0 g/L agar. The medium for the measurement of protease (Prt) is LB solid medium mixed with 4% non-fat milk powder at a ratio of 1:1.

### Establishment of the standard curve of 3-OH PAME

A standard curve of 3-OH PAME was established using 5 different concentrations of 3-OH PAME specimens: 0.25 μM, 0.5 μM, 1 μM, 2 μM and 4 μM (**Supplementary Figure S1**). The correlation between AA values and 3-OH PAME contents were determined. The experiment was repeated in three independent times with four replicates for each concentration each time.

### Isolation and screening of 3-OH PAME QQ strains

The selective-enrichment culture method was used to screen QQ strains (Flagan et al., 2003). Firstly, inter-rhizosphere soil and Casuarina branches were collected from the Haitouwan Casuarina forestry in Zhanjiang, Guangdong Province. The branches were crushed into sawdust, and 10 g of which and the mixed soil sample were respectively added into 200 mL of MSM liquid medium containing 1 μM 3-OH PAME (sole carbon source), shaking at 28°C for 7 d at 200 rpm. After extracting the culture solution in three portions with 30 mL of dichloromethane, the solution was evaporated dried by rotation at 40°C, 500 Pa, eluted in two portions with 1 mL of chromatographic methanol and fixed to 1 mL, 100 μL of which was added into the injection vial containing the interpolation tube and detected by Liquid Chromatograph Mass Spectrometer (LC-MS) with methods described below. The solution with significantly reduced 3-OH PAME content, compared with the solution containing no soil or sawdust sample, was considered as a sample containing 3-OH PAME degrading bacteria.

Single colony bacteria were obtained and purified after streaking the solutions on LB plates. The degradation ability of the isolated strains was determined using the above method.

### LC-MS of 3-OH PAME

LC-MS for quantitative analysis of 3-OH PAME using Q Exactive Focus Liquid chromatography-mass spectrometry (Thermo Fisher, United States) was as follows: chromatographic column: ACQUITY UPLC HSS T3 Column (Waters, United States), flow rate of 0.3 mL/min, temperature of column: 40°C, mobile phase of methanol/water = 85/15, characteristic ion of Na^+^, molecular weight of 309, peak time of 5.5-6.5 min, injection volume of 10 µL.

### Determination of the 3-OH PAME degradation curves of the QQ strains

To investigate the relationship between the bacterial growth and 3-OH PAME degradation, strains Q1-7 and Q4-3 were cultured in LB medium to OD_600_ of 1.2 by shaking at 200 rpm at 28°C, then transferred into MSM+1 μM 3-OH PAME medium at a ratio of 1:100, shaking at 200 rpm at 28°C for 2 d. The MSM+1 μM 3-OH PAME medium added with of LB medium was served as the negative control. Samples were taken every 12 h to measure the concentrations of 3-OH PAME by LC-MS and the OD_600_ values of the QQ strains by UV spectrophotometer. The experiment was repeated in three independent times.

### Genome sequencing, assembly, and annotation

To identify the taxonomic status of the QQ strains, Q1-7 and Q4-3 were grown in LB medium by shaking at 200 rpm at 28°C to OD_600_ of 1.0. The genomic DNAs of both strains were extracted using an EasyPure Bacterial Genomic DNA Kit (Transgen, Beijing, China). DNA qualities were determined by agarose gel electrophoresis and Qubit Fluorometer Quantitation (Thermo Fisher Scientific, Waltham, MA). Genomic DNAs of Q1-7 were sent to Novogene (Tianjin, China), and Q4-3 was sent to Biomarker (Beijing, China) for sequencing. The genome sequence of strain Q1-7 was determined using the Hiseq × Ten PE150 in combination with the PacBio RS II platform. A mixed *de novo* assembly of PacBio long-line and Illumina short-line sequencing data was performed on the genome of Q1-7 using Unicycler (v.0.4.7). The Q1-7 genome was annotated using the Prokaryotic Genome Annotation Pipeline v.2019-05-13. build3740 (Tatusova et al., 2016). AMRFinder v.3.8.4 was used to identify antimicrobial resistance genes (Feldgarden et al., 2020); TXSScan was used to identify secretion systems (Abby and Rocha, 2017); and the tool dbCAN2 was used to identify carbohydrate-active enzymes (Zhang et al., 2018). A sequence search of the MEROPS database was used to identify proteases using the tool BlastP (v.2.9.0, NCBI) (Rawlings et al., 2010). The Signal v.4.1 program was used to detect proteins with signal peptides (Petersen et al., 2011). The genome sequence of strain Q4-3 was determined using the NovaSeq 6000 in combination with the Oxford Nanopore Technologies platform. For genome assembly, the filtered reads were assembled by Canu v1.5 software (Koren et al., 2017), and then Circlator v1.5.5 was used to cyclize genome assembly. For genome component prediction, Coding genes prediction was performed by Prodigal v2.6.3 (Hyatt et al., 2010). Transfer RNA (tRNA) genes were predicted with tRNAscan-SE v2.0 (Chan and Lowe, 2019), Ribosome RNA (rRNA) genes were predicted with Infernal v1.1.3. Repetitive sequences were predicted using RepeatMasker v4.0.5 (Tarailo Graovac and Chen, 2009).The whole genome sequences of strains Q1-7 and Q4-3 have been uploaded to NCBI under the accession numbers CP116304.1 (PRJNA922361) and CP115820.1 (PRJNA922444), respectively.

### Phylogenetic analysis of strains Q1-7 and Q4-3

Pairwise average nucleotide identity (ANI) values between Q1-7, Q4-3, and all 327 *Pseudomonas* type genomes available in the NCBI RefSeq database (**Table S2**) were calculated using fastANI v1.3. dDDH values between Q1-7, Q4-3, and their highest ANI values with Q1-7 or Q4-3 were then determined by using the GGDC server (http://ggdc.dsmz.de/). Orthologous genomes were constructed from annotated proteins in representative genomes of Q1-7, Q4-3 and 327 *Pseudomonas* species using OrthoFinder v2.3.2. Phylogenetic analysis of the Q1-7, Q4-3 and representative *Pseudomonas* genomes was inferred from the presence of single copy orthologs in most of the genomes analyzed. Single gene alignments were generated using MAFFT v7.490, and spot columns were trimmed using trimAl v1.4 and then concatenated into a supermatrix. Phylogenetic inference was performed using IQ-TREE v2.1.2 to automatically select the most appropriate evolutionary model for each gene, and the reliability of internal branching was estimated by ultra-fast bootstrap analysis with 1000 replicates.

### Phenotypic characteristic analyses of strains Q1-7 and Q4-3

Utilization of carbon sources of strains Q1-7 and Q4-3 were determined using Biolog GEN III microplates (bioMérieux) following the manufacturer’s protocol.

To determine the esterase activity of strains Q1-7 and Q4-3, bacteria were cultured in LB medium by shaking at 28°C at 200 rpm until OD_600_ of 1.2, 1 μL of which was spotted onto the LB agar plates supplemented with 1% or 2% tributyrin and incubated in an incubator at 28°C for 2 d or 4 d (Lee et al., 2004). The diameters of the degradative halos were measured. The experiment was repeated three times.

To test the motility of the strains, Q1-7 and Q4-3 were cultured in LB medium by shaking at 200 rpm at 28°C until OD_600_ of 1.2. Swimming, swarming and twitching media were prepared as described previously. Specifically, 15 mL of the medium was poured in a 90-mm diameter dish, followed by the addition of 1 μL of bacterial culture to the inside of the swimming medium, the surface of the swarming medium and the bottom of the twitching medium, and incubated at 28°C for 24, 36 and 24 h, respectively. The swimming and swarming plates can be photographed and measured directly, while the twitching plates were stained with 2% crystalline violet for 20 min, then rinsed three times with ddH_2_O and then photographed and measured. The diameter of the bacterial movement was measured and collated using Image J 1. 52a. The experiment was repeated three times.

For measurement of the biofilms formed by strains Q1-7 and Q4-3, bacteria were cultured in LB medium by shaking at 200 rpm at 28°C until OD_600_ of 1.2, 1 μL of which was added into 100 μL of SOBG medium in each well of polystyrene 96 well-tissue culture plate (Guangzhou Jet Bio-Filtration Co., Ltd., China). The plate was incubated for shaking at 150 rpm at 28°C for 48 h. The culture medium was discarded and 200 μL of 0.1% crystal violet (wt/vol) was added to the wells for staining. Each well of the 96-well plate was washed three times with water and dried in air, and 250 μL of 95% ethanol was added to dissolve the crystal violet from the stained bacterial biofilm cells. The amount of biofilm formation was quantified by measuring the absorption values at 595 nm on a Multifunctional Microplate Reader (MMR) (Microplate reader, BioTek, United States) (Chen et al., 2013). The experiment was repeated three times.

The antimicrobial susceptibility of the QQ strains was tested to further investigate their resistance (Jorgensen and Ferraro, 2009; Hu et al., 2021). Strains Q1-7 and Q4-3 were cultured in LB medium by shaking at 200 rpm at 28°C until OD_600_ of 1.2, 1 μL of which was added into 100 μL of LB medium in each well of polystyrene 96 well-tissue culture plate (Guangzhou Jet Bio-Filtration Co., Ltd., China). The plate was incubated for shaking at 200 rpm at 28°C for 24 h. Antibiotics including ampicillin, kanamycin, streptomycin, gentamicin, tetracycline, and Polymyxin B were used in this experiment at concentrations of 5, 10, 20, 40, 80, 160, 320, and 640 μg/mL. The minimum antibiotic concentration with no visible cells was defined as MIC. Three replicates were performed for each treatment.

### Measurement of EPS production

The Rdar phenotype, described as the red-dry-rough phenotype, can be used to detect components associated with biofilms such as EPS, cellulose and aggregated hairs (Romling et al., 1998). A final concentration of 0.005% Congo Red was added to the melted YEB medium, mixed and added to a 6-well plate with 3 mL of medium per well. The strains Q1-7 and Q4-3 were cultured in LB medium by shaking at 200 rpm at 28°C until OD_600_ of 1.2. NS25 was cultured in CPG medium by shaking at 200 rpm at 30°C until OD_600_ of 1.2. Every 1 µL of NS25+LB, NS25+Q1-7, NS25+Q4-3, Q1-7+CPG and Q4-3+CPG mixtures (1:1) was inoculated on the center of the well and incubated at 28°C, and photographed every 24 h. The experiment was repeated in triplicate.

In addition, NS25 was incubated with each of the QQ bacteria until OD_600_ to 1.2, NS25+LB, NS25+Q1-7, NS25+Q4-3, Q1-7+CPG and Q4-3+CPG (1:1) were prepared, diluted and spread on the TTC plates and incubated upside down at 28°C. The effect of QQ bacteria on NS25 EPS yield was visualized by the extent and coverage area of EPS around NS25 colonies after 4 d. The experiment was repeated in triplicate.

### Measurement of cell wall degrading enzymatic activities

Cell wall degrading enzyme activity was measured using the medium formulation described above (Zhou et al., 2016; Hu et al., 2021), and 40 mL of the medium was poured into a 130-mm diameter Petri dish, and wells in 5 mm diameter were punched. The strains Q1-7 and Q4-3 were cultured in LB medium by shaking at 200 rpm at 28°C until OD_600_ of 1.2. NS25 was cultured in CPG medium by shaking at 200 rpm at 30°C until OD_600_ of 1.2. Some of the NS25 bacterial culture was centrifuged at 12,000 rpm for 5 min, and the supernatant was filtered through a 0.22 μm filter. To test the cell degrading enzymatic activity of the strains, 20 μL of the bacterial culture(s)/NS25 supernatant was added to the well, and incubated at 28°C for 24 h. Pel and Peh plates were treated with 1 M HCl for 15 min. Cel plates were stained with 0.1% (w/v) Congo red for 15 min and decolored with 1 M NaCl. Prt plates were observed directly without any treatment. The diameter of the transparent halo was measured. To test the NS25 cell numbers in the wells, a concentric circle was punched around the well with an 8-mm diameter hole puncher. The cut medium was mixed thoroughly in 1 mL of CPG liquid medium, diluted in a gradient and applied to CPG plates supplemented with 50 μg/mL Polymyxin to calculate the CFU number of NS25. The experiment was repeated in triplicate.

### Antagonistic assay between QQ strains and NS25

The antagonistic activity of Q1-7 and Q4-3 against NS25 was tested by spot-on-law assay (Li et al., 2020). Firstly, bacterial cultures were grown in LB medium until OD_600_ of 1.2. secondly, 300 μL of NS25 culture was added into 15 mL of 1% agarose (cooled to 50°C), mixed and poured onto the surface of 15 mL LB agar plates (9 cm × 9 cm), dried at room temperature, and then punched with a 5-mm puncher. Finally, 20 μL of overnight antagonistic candidate cultures were added to the wells. Plates were incubated at 28°C for 24 h. Antagonistic activity was assessed according to the size of the hyaline inhibition circle. The experiment was repeated in triplicate. the antimicrobial activity of NS25 strain against Q1-7 and Q4-3 was also determined using the above method.

### RNA purification, RNA-seq and RT-qPCR

Single colonies of strains Q1-7, Q4-3 and NS25 were grown until OD_600_ of 1.2, and NS25+LB (1:1), NS25+Q1-7 (1:1) or NS25+Q4-3 (1:1) was added to 10 mL of CPG medium at a ratio of 1:50 for 7 h at 28°C for RNA extraction. RNA was extracted using the SV Total RNA Isolation System Kit (Promega, Madison, WI, USA) and further purified using the RNA Cleanup Kit (Qiagen, Hilden, Germany). DNA contamination was eliminated with DNase I. RNA purity and quality were checked by gel electrophoresis and NanoDrop 2000c (Thermo Fisher Science, Waltham, MA, USA). For RT-PCR analysis, 0.5 μg of RNA was reverse transcribed using the HiScript III 1st Strand cDNA Synthesis Kit (Vazyme Biotech Co., Nanjing, China) to generate template cDNA. Expression of the NS25 internal reference gene *infB* was selected to balance the cDNA concentrations in the three samples. The primers used for PCR amplification were listed in **Table S4**. RT-PCR was performed using 2× Rapid Taq Master Mix (Vazyme Biotech Co., Nanjing, China) with the following cycling pattern. 1 cycle at 95°C for 3 min; followed by 40 cycles at 95°C for 15 s, 56°C for 15 s, and 72°C for 15 s; 1 cycle at 72°C for 5 min; and kept at 16°C. The expression of each gene was determined by measuring the signal intensity of the bands using Image Lab software (Bio-Rad, Hercules, CA, USA). Experiments were performed in triplicate.

### Measurement of bacterial growth curves

Single colonies of strains NS25, NS25(*ΔphcB*), NS25(*ΔsolI*) were grown until OD_600_ of 1.2, and diluted into fresh CPG medium in 1:100 ratio. Bacteria were grown with shaking at 200 rpm under 30°C, and cell density was measured at 0, 6, 12, 24, 30, 36, 42, 48, 54, and 60 h respectively. The experiment was repeated three times in triplicate.

### Pathogenicity tests

To evaluate the efficacies of strains Q1-7 and Q4-3 on biocontrolling the bacterial wilt disease, one-year old *Casuarina*, one-month old tomato and peanut seedlings were used for the pathogenicity tests. Strains Q1-7 and Q4-3 were cultured in LB medium by shaking at 200 rpm at 28°C until OD_600_ of 1.2. Strain NS25 was cultured in CPG medium by shaking at 200 rpm at 30°C until OD_600_ of 1.2. For testing the pathogenicity on Casuarina, root-drenching method was used by pouring 10 mL of NS25+Q1-7 (1:1) or NS25+Q4-3 (1:1) into the soil close to the plant roots (Kanda et al., 2003). For testing the pathogenicity on tomato and peanut seedlings, 200 µL of NS25+Q1-7 (1:1) or NS25+Q4-3 (1:1) was injected into the center of the tomato or peanut pseudostem using 1.0 mL needleless syringes. CPG+LB broth without bacteria, and CPG+Q1-7/Q4-3 were served as the blank and negative controls, respectively, and equal volume of NS25+LB was set as the positive control. Inoculated plants were placed in a cabinet with a 14-h light/10-h dark cycle at 28°C. The experiment was repeated three times with twelve seedlings each time. All the plants were monitored for disease analysis, which was set as: survival (%) = (1 - diseased plant number/total plant number) × 100%.

For measurement the NS25 CFU in the plants, each three tomato seedlings were inoculated with 10 mL of CPG+LB, NS25+LB, NS25+Q1-7, NS25+Q4-3, Q1-7+CPG and Q4-3+CPG bacterial cultures, respectively, by irrigation into the pots with some of the roots cut off. After 7 days post inoculation (dpi), 0.5 g of plant roots or stems were cut and washed with sterile water three times, homogenized with 1 mL of ddH_2_O and diluted in series. Dilutions were spread onto TTC plates. The number of NS25 CFU was counted after 3 days. Three independent tests were performed.

### Statistical analysis

The statistical analysis was performed using GraphPad Prism (San Diego, CA). The results were analyzed by Student’s *t*-test, where * indicates *p* < 0.05, ** indicates *p* < 0.01, *** indicates *p* < 0.001, and **** indicates *p* < 0.0001.

## Results

### QQ strains with high degrading ability of 3-OH PAME were obtained from the original habitat of *C. equisetifolia*

In order to directionally cultivate QQ strains in original habitats for the control of bacterial wilt disease, *C. equisetifolia* branches and forest soil were collected from *Casuarina* planting forest in Haitouwan Forest in Zhanjiang city, Guangdong province. MSM medium with 3-OH PAME as the sole carbon source was used to enrich QQ candidate bacteria. Firstly, a standard curve of 3-OH PAME concentrations was established using a serial dilution of 3-OH PAME that generates an equation as Y = 1267805180 × X + 77323744 with a high R^2^ as 0.9973, where Y represents the concentration of 3-OH PAME, X represents the mean AA numerical value of LC-MS (**Supplementary Figure S1**). Secondly, crushed branches and soil samples were respectively added into the MSM+3-OH PAME medium, and cultures with significantly reduced concentration of 3-OH PAME were selected for bacterial separation. Subsequently, single bacterial colony was added into the MSM+3-OH PAME medium and the concentration of 3-OH PAME was measured. As a result, 11 strains with degrading activity of 3-OH PAME were obtained, including 4 strains (Q1-4, Q1-7, Q1-8 and Q1-9) isolated from the forest soil and the other 7 isolated from *C. equisetifolia* branches (**Supplementary Figure S2A**). Among these strains, strain Q1-7 and strain Q4-3 showing high 3-OH PAME degradation rates over 85% were selected for further experimental study (**Supplementary Figures S2A, B**).

**Figure 1:**
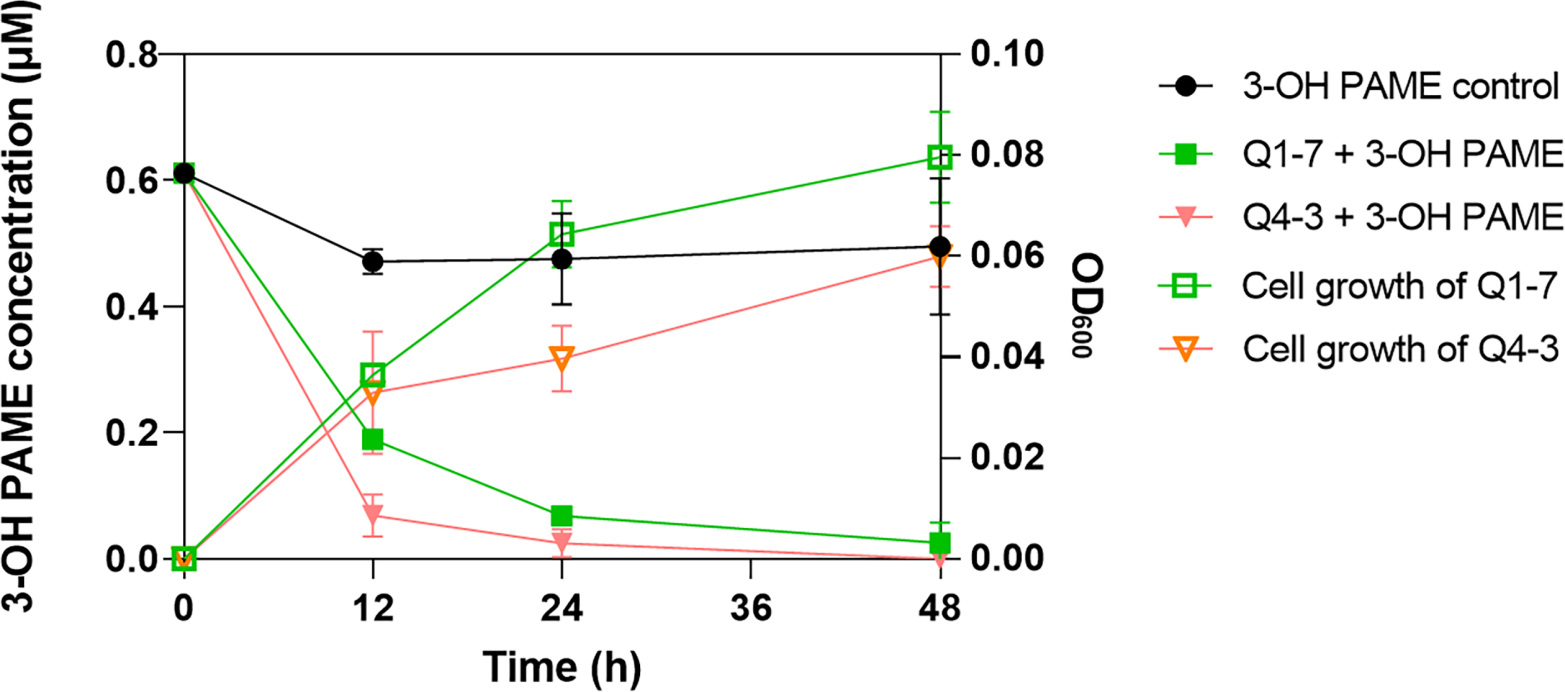
Degradation of 3-hydroxypalmitic acid methyl ester (3-OH PAME) during the growth of strains Q1-7 and Q4-3.

To determine the 3-OH PAME degradation efficiency of Q1-7 and Q4-3 strains, bacterial cultures grown in MSM with 1 µM 3-OH PAME were sampled at different time points for measuring the residual 3-OH PAME concentrations and corresponding cell densities. At 12 hours post inoculation (hpi), the degradation rate of 3-OH PAME was 69.03% by strain Q1-7, and 88.73% by strain Q4-3; at 24 hpi, 88.78% and 95.97% of 3-OH PAME was respectively degraded by strains Q1-7 and Q4-3; at 48 hpi, 95.80% of 3-OH PAME was essentially degraded by strain Q1-7, and 100% by strain Q4-3 (**Figure 1**). At time points of 12 hpi, 24 hpi and 48 hpi, the corresponding OD_600_ of strain Q1-7 was 0.037, 0.064, and 0.080, respectively, and which of strain Q4-3 was 0.033, 0.040, and 0.060, respectively (**Figure 1**). The results showed that the concentrations of 3-OH PAME in the solution dramatically decreased at the first 24 h with bacteria gradually grew (**Figure 1**), suggesting that strains Q1-7 and Q4-3 could degrade 3-OH PAME by using it as a carbon source for bacterial growth.

### Genome sequencing of strains Q1-7 and Q4-3 reveals Q1-7 is a novel species of *Pseudomonas*, and Q4-3 is *Pseudomonas tohonis*

**Figure 2:**
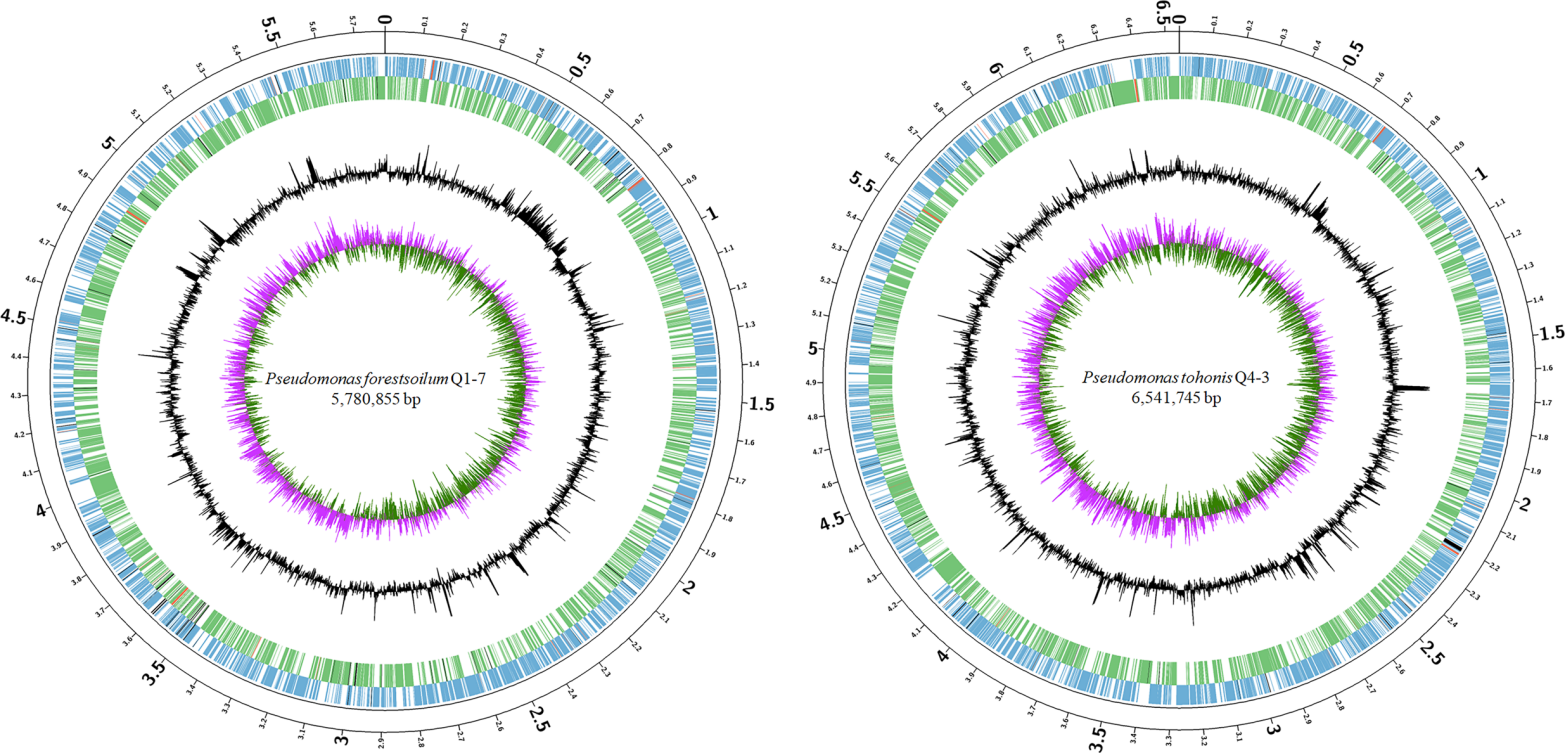
Circular chromosome map of *Pseudomonas forestsoilum* sp. nov. strain Q1-7 and *Pseudomonas tohonis* Q4-3. The circles (from outside to inside) represent features of the positive strand, showing coding sequence (CDS) (blue), rRNA (red), and pseudogenes (black); features of the negative strand, showing CDS (green), rRNA (red), and pseudogenes (black); GC content; and GC-skew (pink and green indicate values higher and lower than the mean value, respectively).

To better understand the molecular basis of the 3-OH PAME quenching ability, the genomes of Q1-7 and Q4-3 were sequenced using both Nanopore PromethION platform and Illumina NovaSeq platform. The genomes of Q1-7 and Q4-3 were both assembled into single, circular chromosomes of 5,780,855 bp and 6,541,745 bp in sizes, with 64.77% and 66.97% in GC-content, respectively (**Figure 2**; **Supplementary Table S1**). A total of 5,146 and 5,826 protein-coding genes, 84 and 167 RNA genes, both 12 rRNA genes, 101 and 15 transposases, and 97 and 51 pseudogenes were respectively predicted in the genomes of Q1-7 and Q4-3 (**Supplementary Table S1**).

To determine the taxonomic classifications of Q1-7 and Q4-3, we compared their genomes with 327 *Pseudomonas* type genomes available in the NCBI RefSeq database, which altogether represent all the known *Pseudomonas* species. We first calculated the Average Nucleotide Identity (ANI) values between all genomes. The results showed that Q1-7 is most closely related to *Pseudomonas lalkuanensis* PE08 (GCF_008807375.1, isolated from contaminated soil collected from a paper mill yard in Lalkuan, Uttarakhand, India) with an ANI value of 88.5638% (**Supplementary Table S2**) and a dDDH value of 33.30%, both below the commonly accepted thresholds (ANI ≥ 95%, and dDDH ≥ 70%) for species delineation. Therefore, we proposed to name the novel species represented by Q1-7 as *Pseudomonas forestsoilum* sp. nov. after the species name of its isolated substrate. For Q4-3, the closest relative is *Pseudomonas tohonis* TUM18999 (AP023189.1, isolated from the skin of a patient with burn wounds in Japan) with an ANI value of 97.8949% (**Supplementary Table S2**) and a dDDH value of 81.90%, indicating that Q4-3 is classified as *P. tohonis*.

**Figure 3:**
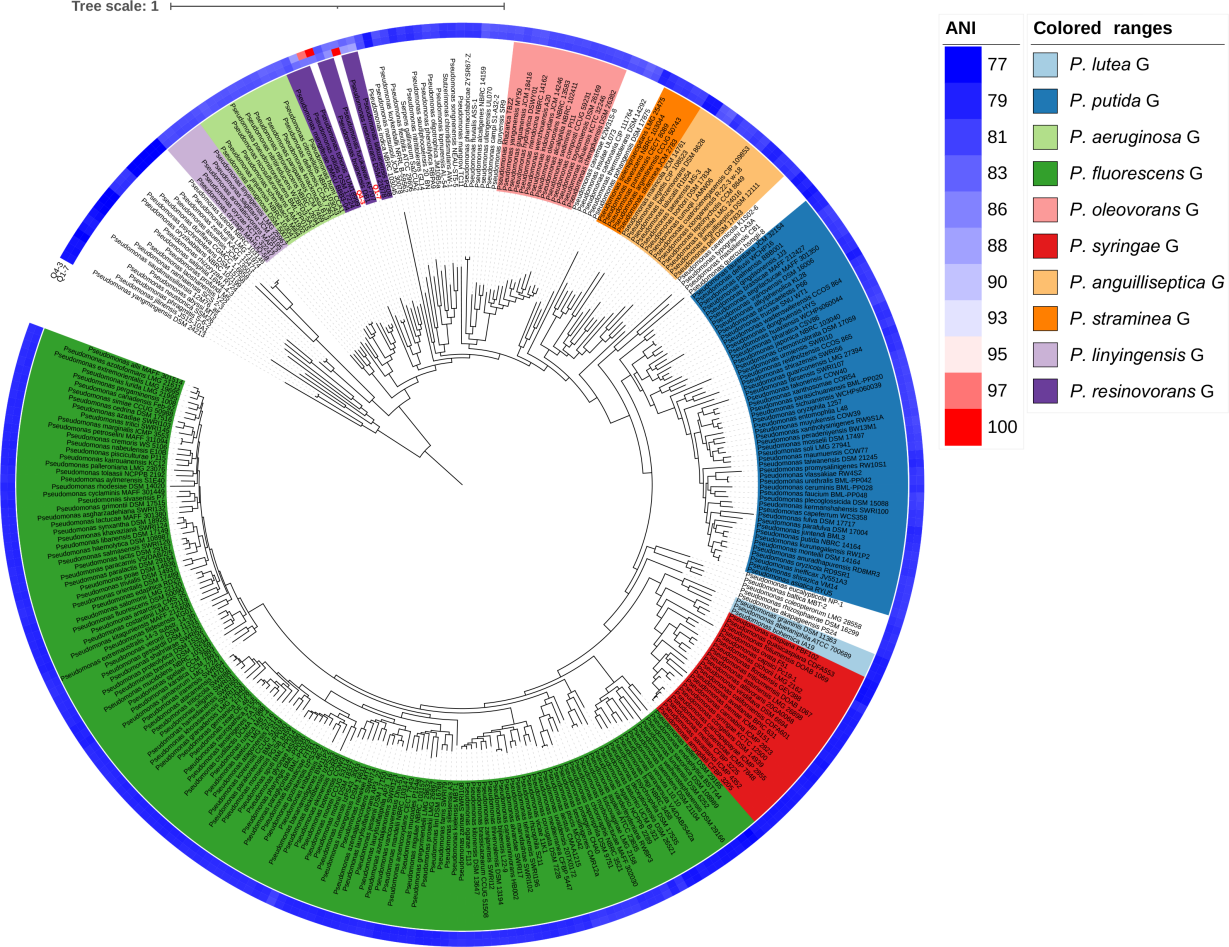
Phylogenetic tree based on 120 single-copy orthologous genes between *Pseudomonas forestsoilum* sp. nov. strain Q1-7 genome*, Pseudomonas tohonis* Q4-3 genome and 327 type strains of other *Pseudomonas* spp. genomes in NCBI database. The closest strain of Q1-7 is *P. lalkuanensis* PE08, with an 88.5638% ANI value, and the closest strain of Q4-3 is *P. tohonis* TUM18999, with a 97.8749% ANI value.

In order to visualize the evolution of Q1-7 and Q4-3 in the *Pseudomonas* genus, we constructed a phylogenetic tree based on 120 single-copy orthologous genes between each of them and 327 type strains of the *Pseudomonas* to represent their relationships. The phylogenetic tree based on whole genome sequences showed that Q1-7 and Q4-3 both belong to the *Pseudomonas resinovorans* group (**Figure 3**), and Q1-7 is over 88% identical to its sister branch including *P. lalkuanensis* PE08 and *P. resinovorans* DSM 21078, suggesting that Q1-7 represents a new species. Q4-3 is closest to *P. tohonis* TUM18999, locating on the same branch, indicating that strain Q4-3 is *P. tohonis*. The evolutionary distance between the two strains is farther apart, consistent with the lower ANI (85.3783%) and dDDH values (26.50%) between them (**Supplementary Table S2**).

### Phenotypic characteristics of strains Q1-7 and Q4-3

In addition, biochemical characteristics of strains Q1-7 and Q4-3 were also tested, which revealed most consistent features between the two strains except that strain Q1-7 could use D-Galacturonic acid and L-Galactonic acid, while strain Q4-3 had a weak response to them; strain Q1-7 could not use D-Melibiose, α-D-Glucose, D-Fructose, L-Lactic acid, L-Alanine and L-Glutamic acid, while strain Q4-3 is weakly responsive to them; strain Q1-7 has a weak response to D-Fucose and Lincomycin, while Q4-3 could use them (**Table 1**).

**Table 1:** Biochemical characteristics of strains Q1-7 and Q4-3.

Utilization of (Biolog GEN III)	Q1-7	Q4-3
Dextrin	W	W
D-Maltose	–	–
D-Trehalose	–	–
D-Cellobiose	–	–
Gentiobiose	–	–
Sucrose	–	–
D-Turanose	–	–
Stachyose	–	–
D-Raffinose	–	–
α-D-Lactose	–	–
D-Melibiose	–	W
β-Methyl-D-glucoside	–	–
D-Salicin	–	–
N-Acetyl-D-glucosamine	–	–
N-Acetyl-β-D-mannosamine	–	–
N-Acetyl-D-galactosamine	–	–
N-Acetyl neuraminic acid	–	–
α-D-Glucose	–	W
D-Mannose	–	–
D-Fructose	–	W
D-Galactose	W	W
3-Methyl glucose	W	W
D-Fucose	W	+
L-Rhamnose	W	W
Pectin	–	–
D-Gluconic acid	–	–
D-Galacturonic acid	+	W
L-Galactonic Acid	+	W
Glucuronamide	+	+
Mucic acid	W	–
Quinic acid	+	+
D-Saccharic acid	–	–
L-Lactic acid	–	W
Citric acid	W	–
α-Keto-Glutaric acid	W	W
D-Malic acid	–	–
L-Malic acid	W	W
Tween 40	–	–
γ-Amino-butryric acid	–	–
Acetoacetic acid	–	–
Formic acid	–	–
Acetic acid	W	W
Propionic acid	–	–
Inosine	–	–
D-Sorbitol	–	–
D-Mannitol	–	–
D-Arabitol	–	–
Glycerol	–	–
D-Fructose-6-PO_4_	W	W
D-Serine	–	–
L-Serine	–	–
L-Alanine	–	W
L-Arginine	–	–
L-Aspartic acid	–	–
L-Glutamic acid	–	W
L-Histidine	–	–
Gelatin	–	–
Glycyl-L-proline	–	–
Troleandomycin	W	W
Rifamycin SV	+	+
Lincomycin	W	+
Vancomycin	+	+

+, -, and W respectively indicate positive, negative, and weak reaction.

**Figure 4:**
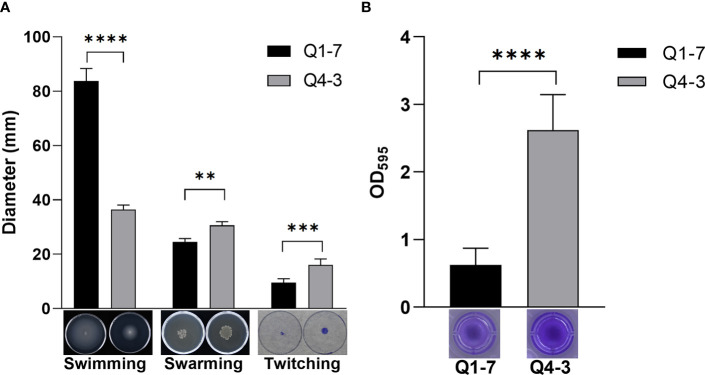
Cell motility, biofilm formation of strains Q1-7 and Q4-3. **(A)** Swimming, swarming and twitching motilities of strains Q1-7 and Q4-3. For the swimming and swarming motilities, 1 μL of bacterial culture (OD_600_ = 1.2 in LB medium) was spotted into the medium or onto the center of a plate containing 15 mL of semisolid swimming or swarming medium, which was then incubated at 28°C for 24 h for swimming and 36 h for swarming before measurement of the diameters of bacterial motility zones. For the twitching motility, 1 μL of bacterial culture (OD_600_ = 1.2 in LB medium) was spotted onto the center of a plate containing 15 mL of semisolid twitching medium, which was then incubated at 28°C for 24 h, then stained with 2% crystal violet (w/v) for 20 min and measured the diameter after rewashing with ddH_2_O. **(B)** Biofilm formation of strains Q1-7 and Q4-3. At a ratio of 1:100, 1 μL of bacterial culture (OD_600_ = 1.2 in LB medium) was added into 100 μL of SOBG medium in the well of polystyrene 96 well-tissue culture plate. The plate was incubated at 28°C at 150 rpm for 48 h, the culture medium was discarded and 150 μL of 0.1% crystal violet (w/v) was added to the wells. The wells were washed three times with water and dried in air, added with 200 μL of 95% ethanol to dissolve the crystal violet. The amount of biofilm formation was quantified by using the absorption value at 595 nm on a Multifunctional Microplate Reader (MMR) (Microplate reader, BioTek, United States). All the assays were repeated three times in triplicate. ** indicates *p* < 0.01, *** indicates *p* < 0.001, and **** indicates *p* < 0.0001.

The motility of bacteria determines their ability to colonize and expand in the plant root zone. To defend the host against pathogenic bacteria, the motility of the biocontrol bacteria can also have an impact on its ability to defend itself (Piromyou et al., 2015; Liu et al., 2017). Thus, we tested the swimming, swarming and twitching motilities of strains Q1-7 and Q4-3 to reflect their colonization and expansion potential in soil rhizosphere or plants. The results showed that strain Q1-7 showed strong swimming and swarming motility, but weak twitching motility, while strain Q4-3 showed stronger swarming and twitching motilities than strain Q1-7 (**Figure 4A**). The significant motility difference between the two strains may be related with their different isolated habitats, where strain Q1-7 was isolated from the forest soil and strain Q4-3 was isolated from *C. equisetifolia* branch.

Biofilm is a membrane-like structure that forms under specific conditions and is formed when bacteria are adsorbed onto a solid surface and then proliferate (Branda et al., 2005). We tested the biofilm of strains Q1-7 and Q4-3 on SOBG medium. The results show that both Q1-7 and Q4-3 strains can form biofilms, with Q1-7 producing much less than Q4-3 (**Figure 4B**).

In addition, we tested the susceptibility of strains Q1-7 and Q4-3 to six different antibiotics. The results showed that they are sensitive to most tested antibiotics except ampicillin, and strain Q4-3 is also resistant to streptomycin with minimal inhibitory concentration (MIC) up to 40 µg/mL (**Supplementary Table S3**).

### Q1-7 and Q4-3 strains have esterolytic activities

**Figure 5:**
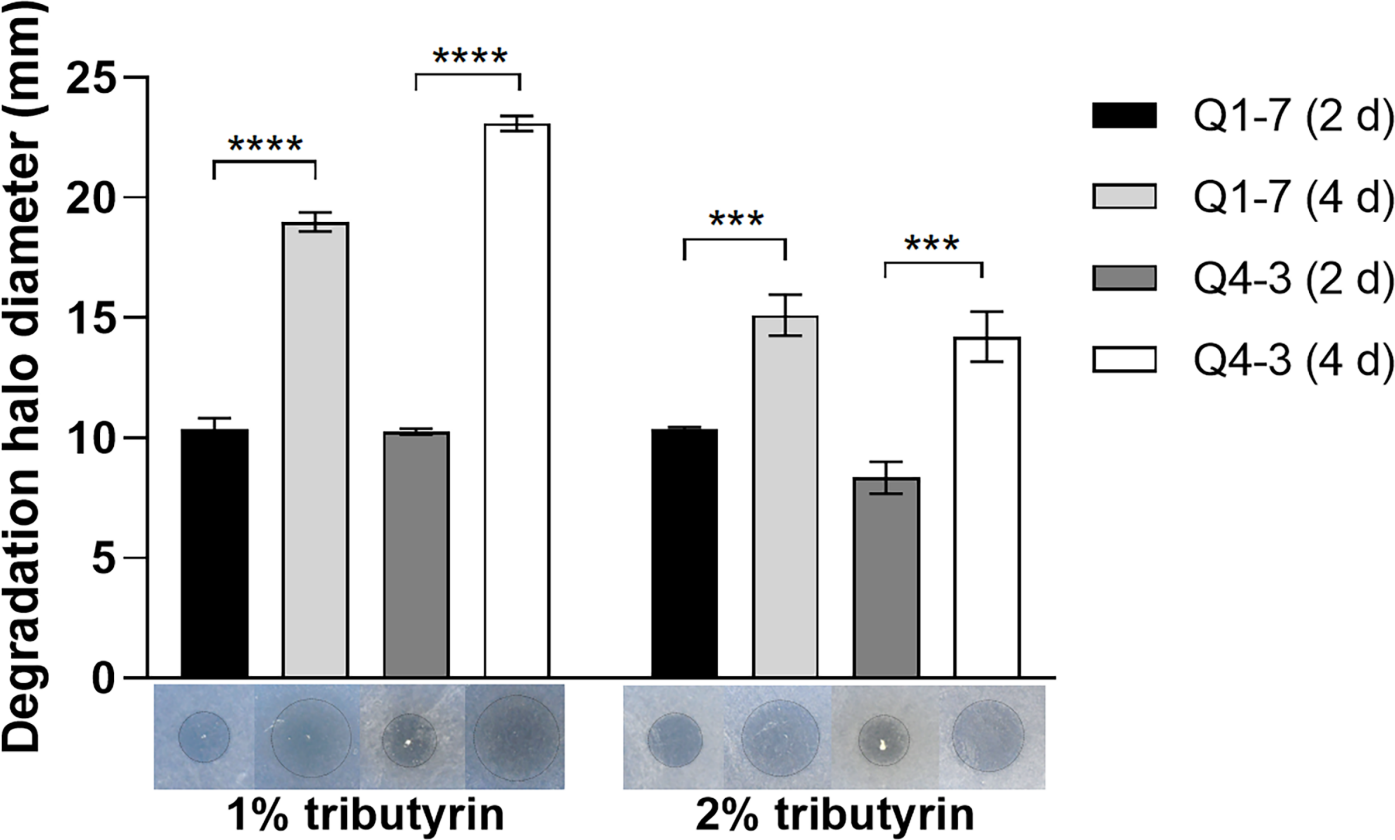
Esterase activity test of strains Q1-7 and Q4-3. The esterase activity was measured on LB plates with 1% and 2% triacylglycerol, and the size of the esterolytic circlea produced on day 2 and day 4 was observed and measured. *** indicates *p* < 0.001, and **** indicates *p* < 0.0001.

The most important signaling molecule in the pathogenic regulatory networks of *R. solanacearum* is 3-OH PAME, the chemical nature of which is an ester compound. To confirm the ability of strains Q1-7 and Q4-3 to degrade esters, the diameters of the degradation halos on the LB plates containing tributyrin were observed to reflect their esterolytic abilities. Degradation halos were produced after the addition of strains Q1-7 and Q4-3 bacterial solution to plates containing tributyrin, indicating that strains Q1-7 and Q4-3 can produce esterase to degrade tributyrin. On the 2^nd^ day, both strains showed comparable esterolytic activities on the plates with 1% and 2% tributyrin, whereas, on the 4^th^ day, both strains had significantly greater degradation activities on the plates with 1% tributyrin than on those with 2% tributyrin (**Figure 5**).

### Q1-7 and Q4-3 strains decrease EPS production and cellulase activity of *R. solanacearum*

**Figure 6:**
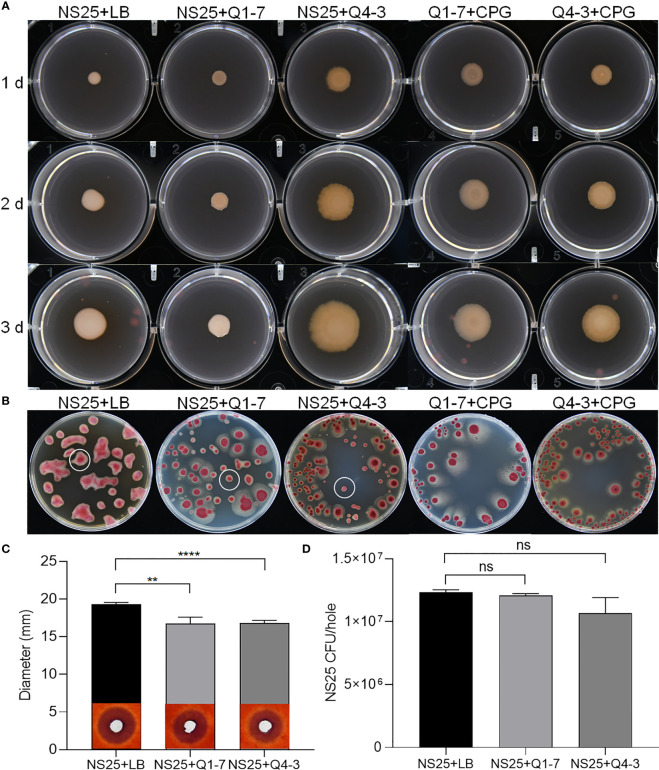
QQ strains reduce the production of EPS and cellulase activity of *R. solanacearum.* **(A)** Each 1 µL of NS25+LB, NS25+Q1-7, NS25+Q4-3, Q1-7+CPG, and Q4-3+CPG (1:1) were inoculated on the surface of the medium, incubated at 30°C and photographed every 24 h **(B)** NS25+LB, NS25+Q1-7, NS25+Q4-3, Q1-7+CPG, and Q4-3+CPG (1:1) were diluted and coated on the TTC plates, and incubated upside down at 30°C. Photos were taken after 4 d **(C)** Cell suspensions of NS25 and QQ strains (Q1-7, Q4-3) at OD_600_ = 1.2 was used. NS25+LB and NS25+QQ were mixed 1:1 and 20 µL of the mixture was added to wells of 5 mm diameter, incubated at 28°C for 24 h, stained with 0.1% (w/v) Congo red, decoloured with 1 M NaCl and photographed to measure the diameter of the transparent circle. **(D)** Before staining with Congo red, the medium around the spiked wells was removed using a large bore punch, diluted with ddH_2_O and applied to TTC+Pb (NS25, but not QQ strains, is resistant to Pb) plates and the number of CFU of NS25 in each well was counted. ** indicates *p* < 0.01, **** indicates *p* < 0.0001, and ns indicates not significant.

*R. solanacearum* NS25, Q1-7 and Q4-3 were all able to produce extracellular polysaccharides (EPS). On YEB plates, the EPS produced by NS25 are smooth and sticky with neat edge, while those produced by Q1-7 and Q4-3 are dry and slightly protruding with irregular edge (**Figure 6A**). After mixing NS25 with either QQ bacteria at a ratio of 1:1, the EPS formed by NS25+Q1-7 were biased towards NS25 with significantly less EPS than that of the NS25+LB group, while those formed by NS25+Q4-3 were biased towards Q4-3 (**Figure 6A**). On TTC plates, the bacterial morphology between NS25 and the two QQ strains could be visually distinguished. NS25 formed very thick and viscous EPS, while Q1-7 and Q4-3 seemed dry and flat. On both NS25+Q1-7 and NS25+Q4-3 group plates, the NS25 colonies formed significantly reduced EPS (**Figure 6B**), suggesting that both Q1-7 and Q4-3 could inhibit the yield of EPS in NS25, with Q4-3 had a stronger inhibitory capacity.

*R. solanacearum* NS25 produced cellulases and polygalacturonases, but not proteases or pectinases (**Supplementary Figure S3A**). Strains Q1-7 and Q4-3 did not produce plant cell wall degrading enzymes except that strain Q4-3 produced proteases (**Supplementary Figure S3A**). There was no significant difference in the cellulase degradation sizes produced by NS25 broth and supernatant, indicating that NS25 hardly grew on cellulase plates (**Supplementary Figure S3B**). Both NS25+Q1-7 and NS25+Q4-3 co-cultures produced significantly less cellulases than the NS25+LB positive control (**Figure 6C**). To test whether such difference is attributed to the lower cell density of NS25 in the co-cultures than that in the positive control, we measured the NS25 cell numbers in the cultures. The results showed that there was no significant difference between the cell numbers of NS25 in the mixed and pure cultures (**Figure 6D**), indicating that both Q1-7 and Q4-3 strains reduced the cellulase activity of NS25. We also measure the antagonistic activities between the pathogenic bacterium NS25 and quorum quenching bacteria Q1-7 and Q4-3, and found that Q1-7 was not antagonistic to the pathogen NS25, while Q4-3 showed slightly visible inhibition activity against NS25 (**Supplementary Figure S4A**), and the NS25 pathogen had no inhibitory activity against either Q1-7 or Q4-3 (**Supplementary Figure S4B**).

**Figure 7:**
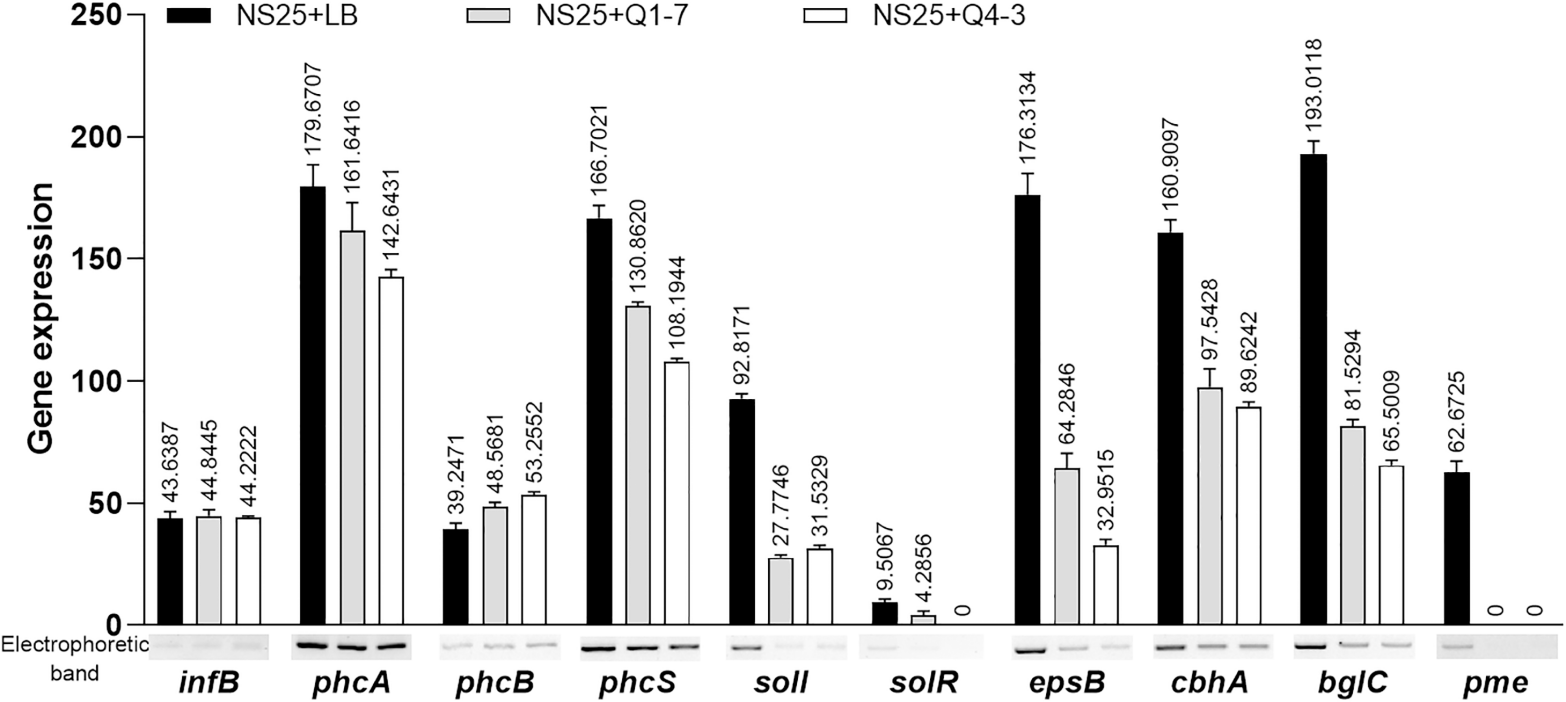
Expression of the genes regulated by the 3-OH PAME in *R. solanacearum* NS25 using RT-PCR. The reference gene *infB* of NS25 was used to equilibrate the concentrations of cDNA samples NS25+LB, NS25+Q1-7 and NS25+Q4-3. The expression of genes was determined by measuring the signal intensity of the bands (under the x axis) using Image Lab software (Bio-Rad, Hercules, CA, USA). Experiments were repeated in triplicate, and the mean data above the bars indicate the signal intensity of RT-PCR bands.

To investigate the consequence of the 3-OH PAME quenching caused by strains Q1-7 and Q4-3, we measured the expression of the *phc* QS regulated downstream genes. The results showed that co-culture of NS25 with Q1-7 or Q4-3 resulted in slightly down expression of the *phc* system (*phcA* and *phcS*) and dramatically down expression of the *sol* system (*solI* and *solR*), and significantly down expression of genes related to EPS and cellulase production (**Figure 7**). This indicates that quenching of 3-OH-PAME caused by strains Q1-7 and Q4-3 could reduce the expression of *phc* regulated genes. Previously, we have obtained the deletion mutants of *phcB* and *solI*. Comparison of the growth curves between NS25, NS25(Δ*phcB*) and NS25(Δ*solI*) revealed that before 24 h, NS25(Δ*phcB*) grew slightly faster than the other two strains, and no obvious difference could be observed between the strains after 24 h (**Supplementary Figure S5**), indicating that these *phc* regulated genes are not closely related to the pathogen growth.

### Q1-7 and Q4-3 strains biocontrol bacterial wilt disease caused by *R. solanacearum*

**Figure 8:**
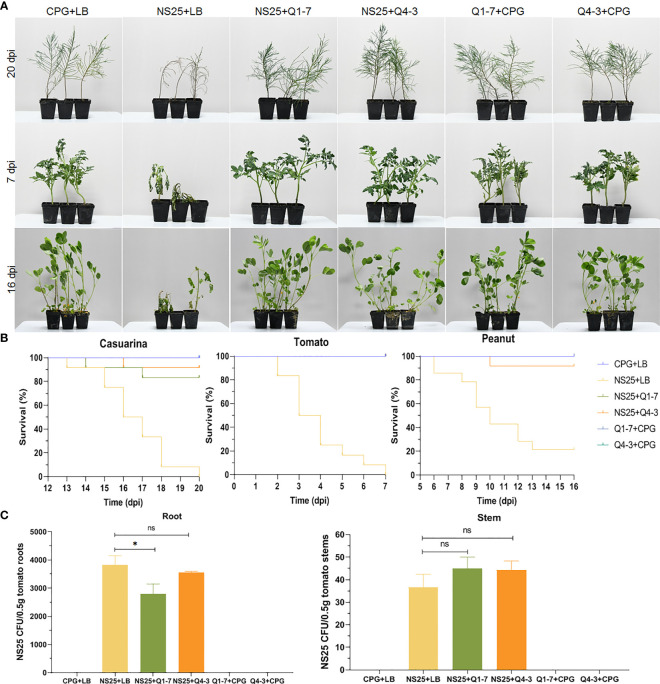
Inhibition of Q1-7, Q4-3 against bacterial wilt disease on *Casuarina*, tomato and peanut. Cell suspensions of *R. solanacearum* NS25 and QQ strains (Q1-7 and Q4-3) at OD_600_ = 1.2 was used. **(A)** Symptoms of the plants inoculated with NS25 and the quenching bacteria. For *Casuarina* seedlings, 10 mL of NS25+QQ bacterial cultures were irrigated into the pots with some of the seedling roots cut off, and 200 μL of NS25+QQ bacterial cultures were injected into the pseudostems of the tomato and peanut seedlings. Twelve tomato, peanut and casuarina seedlings were inoculated each time, and three independent tests were performed with similar results. NS25+LB was used as the positive control, and CPG+LB, Q1-7/Q4-3+CPG were used as the blank and negative controls, respectively. **(B)** The survival curves of the plants inoculated with NS25 and the quenching bacteria. **(C)** Number of NS25 CFU in roots or stems of 0.5 g of tomato plants. The seedlings were inoculated with 10 mL of CPG+LB, NS25+LB, NS25+Q1-7, NS25+Q4-3, Q1-7+CPG and Q4-3+CPG bacterial cultures, respectively, by irrigation into the pots with some of the roots cut off. Three tomato seedlings were inoculated each time and three independent tests were performed. * indicates *p* < 0.05, and ns indicates not significant.

To determine the efficacy of the Q1-7 and Q4-3 strains on controlling or preventing bacterial wilt caused by *R. solanacearum*, three different host plants such as *Casuarina*, peanut, and tomato, were selected for pathogen inoculation. As shown in **Figure 8A**, plants inoculated with *R. solanacearum* NS25 showed symptomatic bacterial wilting, while that inoculated with CPG+LB medium (blank control), NS25+Q1-7, NS25+Q4-3, Q1-7+CPG and Q4-3+CPG (negative controls) kept in a good growth condition. The average survival was calculated based on the daily recorded plant incidence. The results showed that *Casuarina* seedlings inoculated with NS25+LB began to wilt at 15 dpi, and all died at 20 dpi, while only 10% and 20% of the seedlings inoculated with NS25+Q4-3 and NS25+Q1-7, respectively, died at 20 dpi, and all the plants in the blank and negative control groups kept healthy (**Figure 8B**). Tomato seedlings inoculated with NS25+LB began to wilt at 2 dpi and all wilted at 7 dpi, while all the seedlings kept healthy in other treatment groups (**Figure 8B**). Peanut seedlings inoculated with NS25+LB began to wilt at 6 dpi, and 75% died at 16 dpi, while only 10% of the seedlings inoculated with NS25+Q4-3 died at 10 dpi, and all the plants inoculated with NS25+Q1-7, and those in the blank and negative controls kept healthy (**Figure 8B**), suggesting that both strains are effective in prevention and control of plant bacterial wilt caused by *R. solanacearum*, especially for peanut and tomato bacterial wilt.

Calculation of the NS25 cell number in tomato roots and stems indicated that the CFU of NS25 slightly reduced in the roots inoculated with NS25+Q1-7, but was similar in those inoculated with NS25+Q4-3, compared with those in the positive control, and the CFU number in the stems was not obviously different in the NS25+LB, NS25-Q1-7 and NS25+Q4-3 groups (**Figure 8C**), suggesting that the quenching bacteria do not reduce the occurrence of bacterial wilt by inhibiting the growth of NS25.

## Discussion

Quorum quenching (QQ) is a biological control method that attenuates the pathogenicity of pathogenic bacteria by quenching important signaling molecules that play important roles in modulating pathogen virulence (Monteiro et al., 2012; Chen et al., 2013; Ham, 2013; Caicedo et al., 2016). This novel microbial biocontrol strategy has been successfully employed for controlling the diseases of bacterial soft rot caused by *Pectobacterium* through quenching the AHL QS signal (Morohoshi et al., 2009; Kusada et al., 2017), bacterial wilt caused by *R*. *solanacearum* through quenching the AHL QS signal (Chen et al., 2009), Cruciferae black rot caused by *Xanthomonas campestris* pv. *campestris* through quenching the DSF QS signal (Wang et al., 2020; Ye et al., 2020a; Ye et al., 2020b), and bacterial rot of onion bulbs caused by *Burkholderia cenocepacia* through quenching the DSF- and AHL-QS signals (Cui et al., 2019; Wang et al., 2022). In *R*. *solanacearum*, four types of QS signals have been identified, including 3-OH PAME (Flavier et al., 1997), (*R*)-methyl 3-hydroxymyristate [(*R*)-3-OH MAME] (Kai et al., 2015), C6- and C8-homoserine lactone (HSL) (Flavier et al., 1997), and N-(3-hydroxydodecanoyl)-homoserine lactone (3-OH-C12-HSL) (Yan et al., 2022), in which, (*R*)-3-OH MAME is present in *R*. *solanacearum* strains lacking 3-OH PAME (Kai et al., 2015). C6- and C8-HSL appears dispensable for virulence of *R*. *solanacearum* and is under the control of 3-OH PAME (Flavier et al., 1997), and the newly identified 3-OH-C12-HSL signal involved in regulation of cellulase production, motility, biofilm formation, oxidative stress response, and virulence, is present in the phylotypes I, III and IV strains (Yan et al., 2022). Among these QS signals, 3-OH PAME/(*R*)-3-OH MAME is an autoregulator controlling virulence in *R. solanacearum*, controls the expression of EPS, and is the main pathogenic factor in *R. solanacearum* (Clough et al., 1997a; Flavier et al., 1997; Hikichi et al., 2017). In terms of the research progress in 3-OH PAME signal quenching, Shinohara and collaborators screened a 3-OH PAME quenching strain *Ideonella* sp. 0-0013 from soil and purified a protein with 3-OH PAME degrading activity from its supernatant. The amino acid sequence and nucleotide sequence was analyzed and named β-hydroxypalmitoyl methyl ester hydrolase (βHPMEH). The study determined the degradation efficiency of βHPMEH against 3-OH PAME and nine compounds like it, and the inhibitory effect of βHPMEH on the EPS of *R. solanacearum* MAFF 301487, but did not determine the biological control of *Ideonella* sp. 0-0013 and βHPMEH against bacterial wilt (Shinohara et al., 2007). Achari et al. isolated five 3-OH PAME-degrading quenching sterilization strains from the xylem of eggplant and pepper, including *P. aeruginosa* XB7, *P. aeruginosa* XB122, *Rhodococcus corynebacterioides* XB115, *R. corynebacterioides* XB109 and *Stenotrophomonas maltophilia* XB102. In this study, crude enzymes with 3-OH PAME quenching activity were obtained from cell-free supernatant, which inhibited EPS production and cellulase activity of the *R. solanacearum* Rs-09-100, and all five strains of the biocontrol bacteria reduced the incidence of eggplant wilt, but the protein structures or the coding genes of the quenching enzymes were not identified (Achari and Ramesh, 2015). Recently, some proteins including ELP86, ELP96, ELP104, and EstDL33 have been found from a soil metagenome library to degrade 3-OH PAME (Lee et al., 2018).

In this study, we isolated two new 3-OH PAME-degrading bacteria *Pseudomonas forestsoilum* sp. nov. strain Q1-7 and *P. tohonis* Q4-3 which came from the native planting soil and branch habitats of *C. equisetifolia*. Relationship between the degradation rate of 3-OH PAME in MSM medium and the growth status of the two strains revealed that Q1-7 and Q4-3 strains could use 3-OH PAME as the carbon source for growth. The consequence of the signal quenching resulted in reduced production of the major virulence factors produced by *R. solanacearum* NS25, that are cellulases and EPS (Schell, 2000) without affecting the growth of NS25 (**Figure 6**). Accordingly, the expression of Phc-dependent genes, including *phcA*, *phcS*, *solI*, *solR*, EPS encoding genes *epsB*, cellulase encoding genes *cbhA* and *bglC*, and pectin methylesterase encoding gene *pme*, was reduced by the addition of the quenching bacteria (**Figure 7**), suggesting the dominant role of 3-OH PAME in regulating the virulence of *R. solanacearum*. Pathogenicity tests also confirmed the good performance of strains Q1-7 and Q4-3 in biocontrolling the bacterial wilt on *C. equisetifolia*, tomato and peanut (**Figure 8**).

In comparison with strain Q1-7, strain Q4-3 was more effective in the control of *C. equisetifolia* bacterial wilt (**Figure 8B**). It had a stronger degradation activity of 3-OH PAME than Q1-7 although it grew worse in the MSM+3-OH PAME medium (**Figure 1**). On 1% tributyrin plate, Q4-3 also performed better esterolysis than Q1-7 (**Figure 5**). It produces significantly biofilms than Q1-7 (**Figure 4B**), suggesting a stronger ability to attach to plants and resist to external stresses. All these advantages of strain Q4-3 may be attributed to its original habitat in *C. equisetifolia* branch, conferring a better adaptability of the strain in xylem vessels. Comparing the protein sequences of Q1-7 and Q4-3 with the reported sequences of esterases that degrade 3-OH PAME (Shinohara et al., 2007; Lee et al., 2018), the protein sequences obtained were all less than 35% similarity, with a maximum coverage of 97.48% and a minimum of 10.88% (**Supplementary Table S5**), similar to the low homology between the esterases identified by Lee and his colleagues (Lee et al., 2018). This suggests that Q1-7 and Q4-3 do not contain proteins with high homology to the reported esterases, and it is highly probable that the esterases efficiently degrading 3-OH PAME in these two strains have not been reported.

*Pseudomonas* spp. are a relatively common group of plant disease biocontrol strains (Haas and Défago, 2005; Stockwell and Stack, 2007). Apart from our study, there have been only two other *Pseudomonas* strains reported to quench 3-OH PAME so far (Achari and Ramesh, 2015). Actually, many *Pseudomonas* spp. produce secondary metabolites that inhibit the growth of pathogenic microorganisms, such as 2,4-diacetylphloroglucinol (2,4-DAPG), pyoluteorin, pyrrolnitrin, hydrogen cyanide, syringomycin, syringopeptin, viscosinamide, viscosin (Leisinger and Margraff, 1979; Voisard et al., 1989; Sorensen et al., 1996; Raaijmakers et al., 1997; Silby et al., 2011). According to the genomic sequences of strains Q1-7 and Q4-3, the Q1-7 genome has no biosynthetic gene clusters of the secondary metabolites mentioned above, and the Q4-3 genome only contains the synthetic gene cluster of 2,4-DAPG, *phlHGFACBDE*, consistent with the weak antagonistic effect of Q4-3 on NS25 (**Supplementary Figure S5**). Our study indicated that the biocontrol effect of strains Q1-7 and Q4-3 was mainly due to the quenching of 3-OH PAME rather than the inhibition on NS25 growth through the production of antagonistic secondary metabolites. Identification of the quenching genes in strains Q1-7 and Q4-3 will be further studied in future.

## Data availability statement

The datasets presented in this study can be found in online repositories. The names of the repository/repositories and accession number(s) can be found in the article/**Supplementary Material**.

## Author contributions

JZ conceived and designed the experiments. SW isolated and identified the quorum quenching strain and performed the quenching tests. SW, MH, YX, XS, YQ, and FL investigated the control efficiency against bacterial wilts. SW, HC, CL, and XZ analyzed the metagenomic and genome data. SW and JZ wrote and revised the manuscript. All authors contributed to the article and approved the submitted version.
