# Supplementary material for: Pseudomonas forestsoilum sp. nov. and P. tohonis biocontrol bacterial wilt by quenching 3-hydroxypalmitic acid methyl ester
Source: Front Plant Sci. 2023 Jun 30;14:1193297. doi: 10.3389/fpls.2023.1193297 (PMC10349395; doi:10.3389/fpls.2023.1193297)
Supplement: Supplementary file 1 [file DataSheet_1.docx]

***Pseudomonas forestsoilum* sp. nov. and *P. tohonis* Biocontrol Bacterial Wilt by Quenching 3-hydroxypalmitic Acid Methyl Ester**

Si Wang, Ming Hu, Huilin Chen, Chuhao Li, Yang Xue, Xinyue Song, Yuqing Qi, Fan Liu, Xiaofan Zhou, Lian-hui Zhang, Jianuan Zhou*


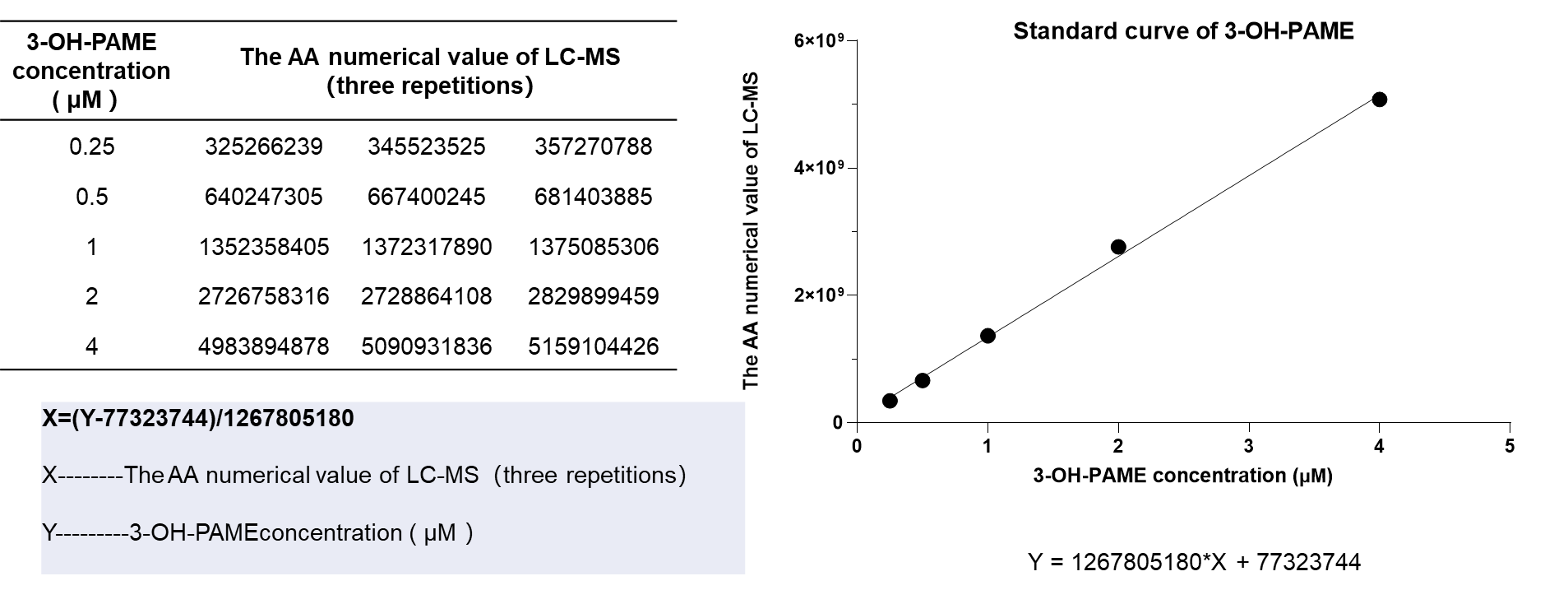


**Figure S1**. Standard curve of 3-OH PAME concentration. The concentration of 3-OH-PAME is calculated by the equation as Y = 1267805180 × X + 77323744, where Y represents the concentration of 3-OH PAME, X represents the mean AA numerical value of LC-MS.


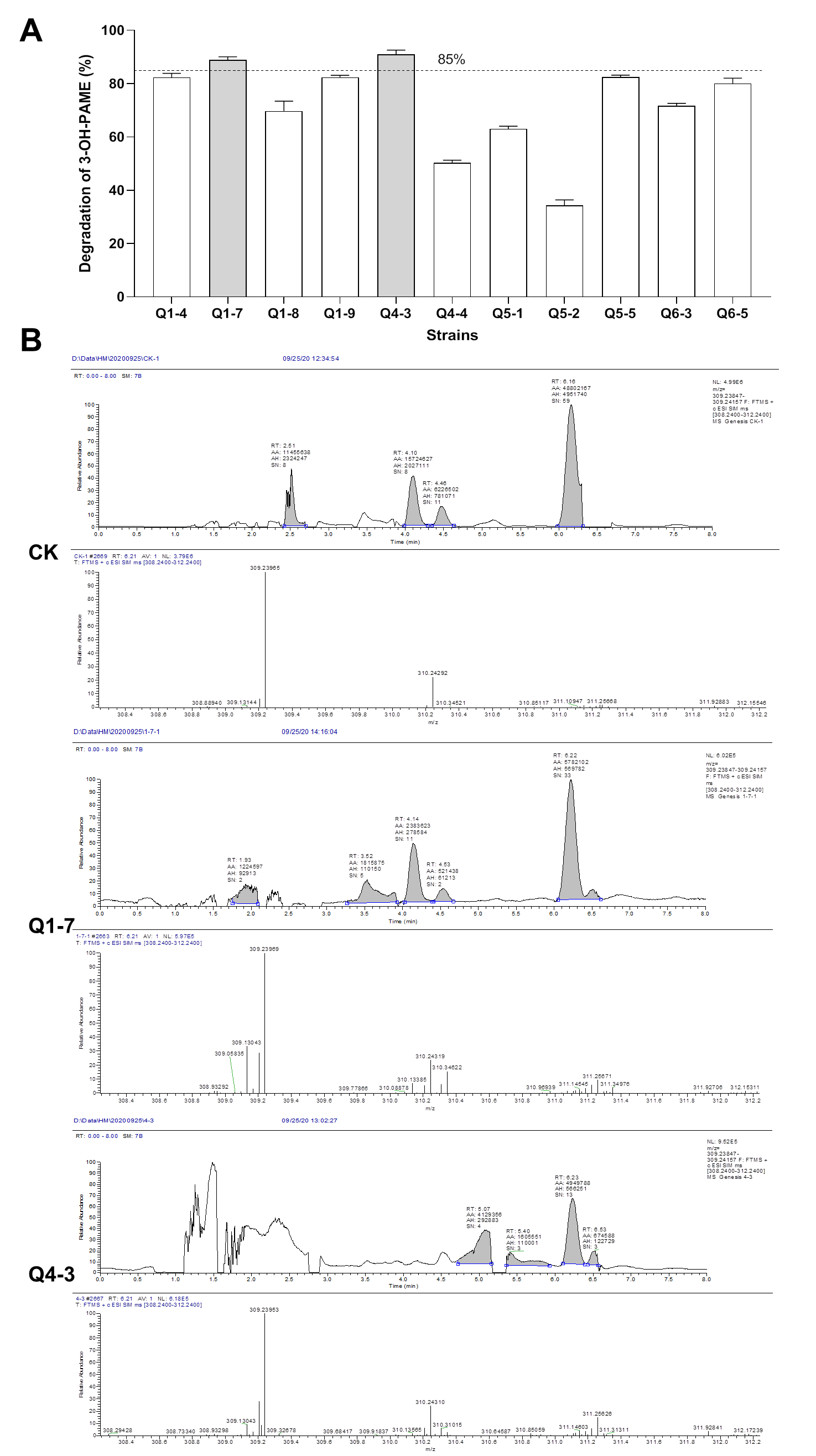


**Figure S2.** Degradation rates of 3-OH-PAME for the bacterial strains isolated from forest soil and *Casuarina* branches from Haitouwan Forest in Zhanjiang city, Guangdong province. (A) Degradation rates of 3-OH-PAME of different strains. (B) Measurement of 3-OH-PAME of strains Q1-7 and Q4-3 using LC-MS.


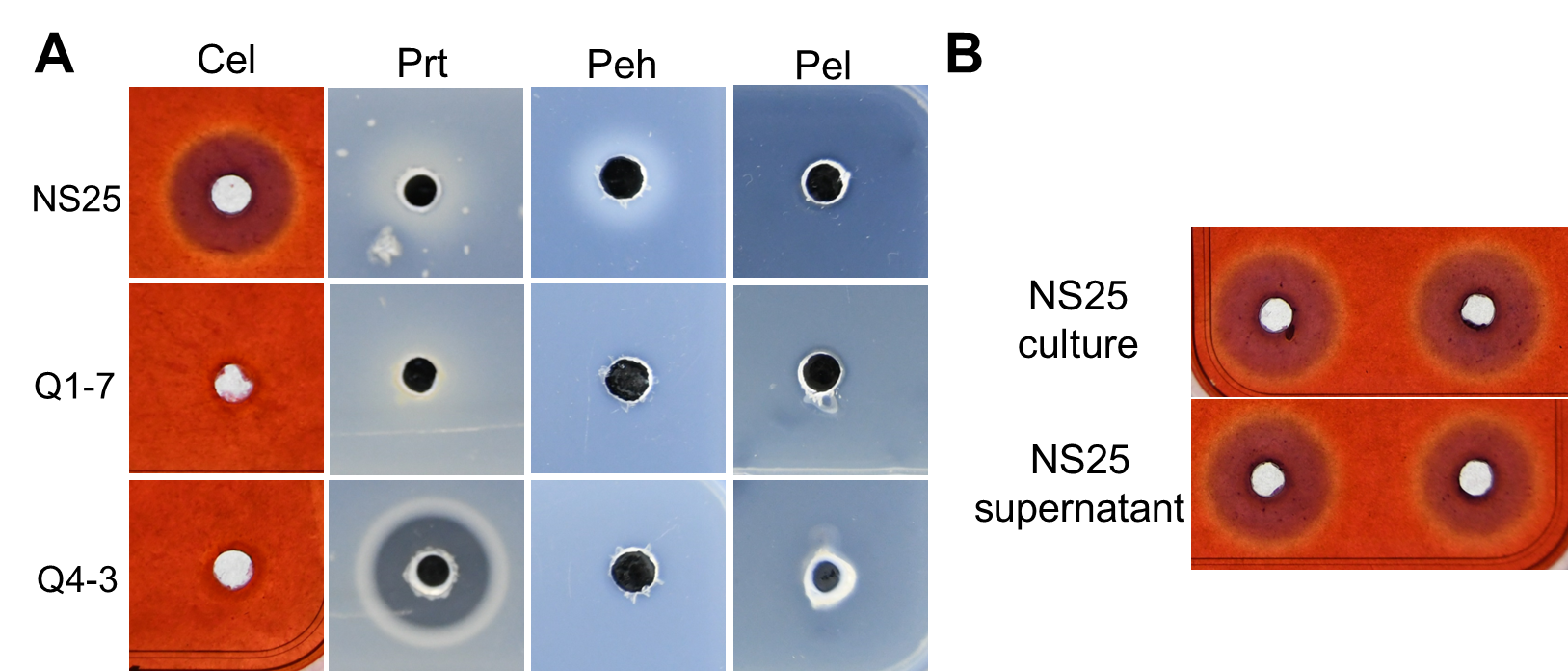


**Figure S3**. Plant cell degrading enzymes produced by NS25, Q1-7 and Q4-3 (A), and the cellulase activity of the NS25 bacterial culture and supernatant (B).


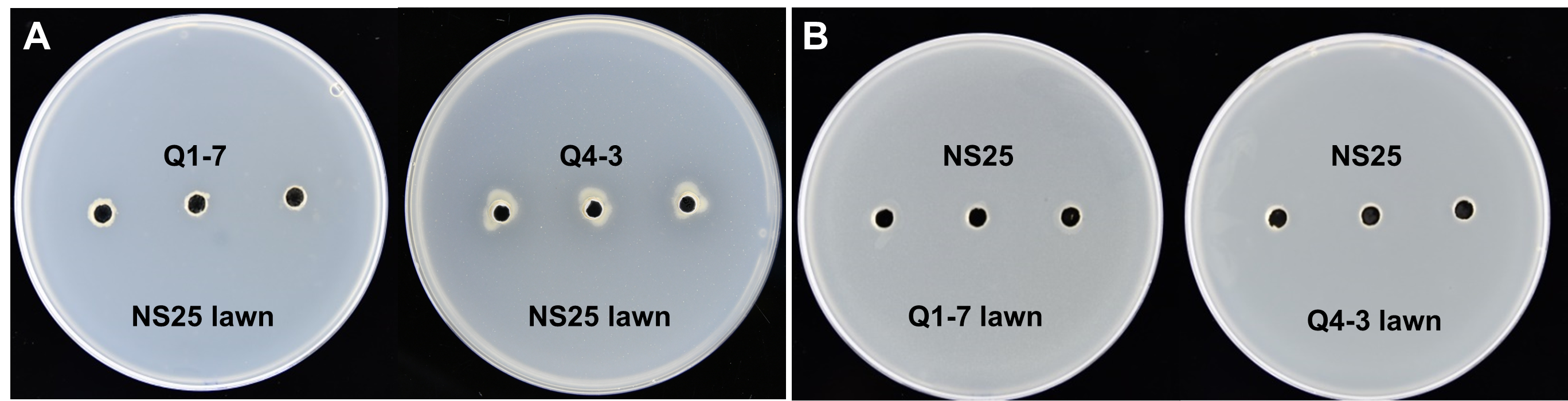


**Figure S4**. Bacteriostatic effects of the quorum quenching bacteria and the pathogenic bacterium NS25 on each other. (A) Antagonistic activity of strains Q1-7 and Q4-3 against the pathogenic strain NS25; (B) Antagonistic activity of the pathogenic strain NS25 against quorum quenching strains Q1-7 and Q4-3.

**Figure S5**. Growth curves of NS25, NS25(ΔphcB) and NS25(ΔsolI) in CPG medium.

**Table S1.** Genomic features of strains Q1-7 and Q4-3

| **feature** | **Q1-7** | **Q4-3** |
| --- | --- | --- |
| Size (bp) | 5,780,855 | 6,541,745 |
| GC content (%) | 64.77 | 66.97 |
| Gene | 5,294 | 6,001 |
| CDS | 5,146 | 5,826 |
| RNA genes | 84 | 167 |
| rRNA | 12 | 12 |
| tRNA | 68 | 65 |
| ncRNA | 4 | 89 |
| Transposase | 101 | 15 |
| Pseudogene | 97 | 51 |
| CRISPR | 2 | 8 |

**Table S2.** The Average Nucleotide Identity (ANI) values between *Pseudomonas forestsoilum* nov. sp. strain Q1-7 genome, *P. tohonis* Q4-3 genome and the *Pseudomonas* genomes in NCBI database

| Species names | Subject genome | ANI value with Q1-7 (%) | ANI value with Q4-3 (%) |
| --- | --- | --- | --- |
| Q4-3 | CP115820.1 | 85.3783 | 100 |
| *Pseudomonas tohonis* TUM18999 | GCF_012767755.2 | 85.4044 | 97.8949 |
| *Pseudomonas otitidis* DSM 17224 | GCF_900111835.1 | 84.8661 | 88.0275 |
| *Pseudomonas lalkuanensis* PE08 | GCF_008807375.1 | 88.5638 | 85.1714 |
| *Pseudomonas furukawaii* KF707 | GCF_000262065.2 | 87.1132 | 85.1577 |
| *Pseudomonas delhiensis* CCM 7361 | GCF_900099945.1 | 83.5573 | 84.3256 |
| *Pseudomonas resinovorans* DSM 21078 | GCF_000423545.1 | 88.0001 | 84.2894 |
| *Pseudomonas carbonaria* CIP 111764 | GCF_904061905.1 | 83.4917 | 84.2298 |
| *Pseudomonas humi* CCA1 | GCF_001748265.1 | 83.6738 | 84.1551 |
| *Pseudomonas citronellolis* NBRC 103043 | GCF_002091555.1 | 83.4775 | 84.0719 |
| *Pseudomonas lalucatii* R1b54 | GCF_018398425.1 | 83.2265 | 83.9035 |
| *Pseudomonas alcaligenes* NBRC 14159 | GCF_000467105.1 | 83.3697 | 83.8927 |
| *Pseudomonas jinjuensis* NBRC 103047 | GCF_002091655.1 | 83.3722 | 83.6526 |
| *Pseudomonas cavernae* K2W31S-8 | GCF_003595175.1 | 82.7196 | 83.4804 |
| *Pseudomonas thermotolerans* DSM 14292 | GCF_000364625.1 | 82.9307 | 83.4074 |
| *Pseudomonas benzenivorans* DSM 8628 | GCF_900100495.1 | 82.8711 | 83.3957 |
| *Pseudomonas knackmussii* B13 | GCF_000689415.1 | 82.9285 | 83.3889 |
| *Pseudomonas ullengensis* UL070 | GCF_014174475.1 | 82.5496 | 83.3285 |
| *Pseudomonas nitritireducens* WZBFD3-5A2 | GCF_010994165.1 | 82.796 | 83.3101 |
| *Pseudomonas campi* S1-A32-2 | GCF_013200955.2 | 82.5555 | 83.2886 |
| *Pseudomonas insulae* UL073 | GCF_016901015.1 | 82.4948 | 83.2618 |
| *Pseudomonas hydrolytica* DSWY01 | GCF_021495345.1 | 82.7721 | 83.2439 |
| *Pseudomonas mangiferae* DMKU BBB3-04 | GCF_007109405.1 | 82.6818 | 83.1993 |
| *Pseudomonas nitroreducens* NBRC 12694 | GCF_002091755.1 | 82.5987 | 83.1903 |
| *Pseudomonas khazarica* TBZ2 | GCF_004521985.1 | 82.4687 | 83.1764 |
| *Pseudomonas guryensis* SR9 | GCF_014164785.1 | 82.3022 | 83.1023 |
| *Pseudomonas guguanensis* JCM 18416 | GCF_900104265.1 | 82.6126 | 83.0921 |
| *Pseudomonas schmalbachii* Milli4 | GCF_017589465.1 | 82.901 | 83.0907 |
| *Pseudomonas boanensis* DB1 | GCF_018704125.1 | 84.3437 | 83.0827 |
| *Pseudomonas pseudonitroreducens* BML-PP015 | GCF_021603645.1 | 82.4731 | 83.0477 |
| *Pseudomonas panipatensis* CCM 7469 | GCF_900099785.1 | 82.6431 | 83.0419 |
| *Pseudomonas nicosulfuronedens* LAM1902 | GCF_005877905.1 | 82.4386 | 82.9612 |
| *Pseudomonas aeruginosa* DSM 50071 | GCF_001042925.1 | 82.4958 | 82.8003 |
| *Pseudomonas kuykendallii* NRRL B-59562 | GCF_900106975.1 | 81.8778 | 82.7854 |
| *Pseudomonas indica* NBRC 103045 | GCF_002091635.1 | 82.4736 | 82.7535 |
| *Pseudomonas oryzae* KCTC 32247 | GCF_900104805.1 | 82.3853 | 82.6953 |
| *Pseudomonas linyingensis* LMG 25967 | GCF_900109175.1 | 82.079 | 82.566 |
| *Pseudomonas oligotrophica* JM10B5a | GCF_021726475.1 | 81.728 | 82.4944 |
| *Pseudomonas oryzagri* MAHUQ-58 | GCF_020831405.1 | 82.2145 | 82.4784 |
| *Pseudomonas sagittaria* JCM 18195 | GCF_900115715.1 | 82.1656 | 82.473 |
| *Pseudomonas indoloxydans* JCM 14246 | GCF_003052605.1 | 81.8555 | 82.418 |
| *Pseudomonas alcaliphila* NBRC 102411 | GCF_002091495.1 | 81.9353 | 82.4163 |
| *Pseudomonas sihuiensis* KCTC 32246 | GCF_900106015.1 | 82.1291 | 82.409 |
| *Pseudomonas chengduensis* DSM 26382 | GCF_012986985.1 | 81.9113 | 82.3507 |
| *Pseudomonas wenzhouensis* A20 | GCF_021029445.1 | 81.9504 | 82.3444 |
| *Pseudomonas aromaticivorans* MAP12 | GCF_019097855.1 | 81.8937 | 82.3019 |
| *Pseudomonas toyotomiensis* DSM 26169 | GCF_002741095.1 | 81.7139 | 82.2974 |
| *Pseudomonas peradeniyensis* BW13M1 | GCF_014268935.2 | 81.8438 | 82.289 |
| *Pseudomonas mendocina* NBRC 14162 | GCF_000813265.1 | 81.8993 | 82.2594 |
| *Pseudomonas yangonensis* MY50 | GCF_009932725.1 | 82.1264 | 82.2531 |
| *Pseudomonas oleovorans* NBRC 13583 | GCF_002091815.1 | 81.954 | 82.234 |
| *Pseudomonas composti* CCUG 59231 | GCF_900115475.1 | 81.7112 | 82.1484 |
| *Pseudomonas ekonensis* COR58 | GCF_019145435.1 | 81.5218 | 82.1277 |
| *Pseudomonas borbori* DSM 17834 | GCF_900115555.1 | 81.9036 | 82.1073 |
| *Pseudomonas japonica* NBRC 103040 | GCF_000730585.1 | 81.6847 | 82.0459 |
| *Pseudomonas faucium* BML-PP048 | GCF_013373935.1 | 81.4512 | 82.0453 |
| *Pseudomonas muyukensis* COW39 | GCF_019139535.1 | 81.4242 | 82.0449 |
| *Pseudomonas parasichuanensis* BML-PP020 | GCF_021601385.1 | 81.3421 | 82.0196 |
| *Pseudomonas sediminis* PI11 | GCF_002741105.1 | 81.5796 | 81.9971 |
| *Pseudomonas tumuqii* LAMW06 | GCF_013184545.1 | 81.7902 | 81.979 |
| *Pseudomonas entomophila* L48 | GCF_000026105.1 | 81.493 | 81.9616 |
| *Pseudomonas oryziphila* 1257 | GCF_003940825.1 | 81.328 | 81.9328 |
| *Pseudomonas xionganensis* R-22-3 w-18 | GCF_009763245.1 | 81.6499 | 81.9186 |
| *Pseudomonas mosselii* DSM 17497 | GCF_000621225.1 | 81.3286 | 81.9164 |
| *Pseudomonas argentinensis* CCUG 50743 | GCF_008801645.1 | 81.1955 | 81.8912 |
| *Pseudomonas flexibilis* ATCC 29606 | GCF_000802425.1 | 81.6175 | 81.8785 |
| *Pseudomonas lopnurensis* AL-54 | GCF_015070855.1 | 81.5281 | 81.8768 |
| *Pseudomonas xantholysinigenes* RW9S1A | GCF_014268885.2 | 81.3958 | 81.8258 |
| *Pseudomonas palmensis* BBB001 | GCF_017848315.1 | 81.1736 | 81.8085 |
| *Pseudomonas straminea* JCM 2783 | GCF_900112645.1 | 81.4574 | 81.8066 |
| *Pseudomonas maumuensis* COW77 | GCF_019139675.1 | 81.4031 | 81.8012 |
| *Pseudomonas qingdaonensis* JJ3 | GCF_002806685.1 | 81.1291 | 81.7978 |
| *Pseudomonas fakonensis* COW40 | GCF_019139895.1 | 81.2774 | 81.7949 |
| *Pseudomonas sichuanensis* WCHPs060039 | GCF_003231305.1 | 81.3362 | 81.7859 |
| *Pseudomonas xanthosomae* COR54 | GCF_019139835.1 | 81.3595 | 81.7577 |
| *Pseudomonas bharatica* CSV86 | GCF_000319305.2 | 81.2349 | 81.6685 |
| *Pseudomonas piscis* MC042 | GCF_009380155.1 | 80.9743 | 81.6261 |
| *Pseudomonas soli* LMG 27941 | GCF_900110655.1 | 81.2443 | 81.6008 |
| *Pseudomonas mangrovi* TC11 | GCF_003052585.1 | 80.9194 | 81.5911 |
| *Pseudomonas plecoglossicida* DSM 15088 | GCF_000688275.1 | 81.1802 | 81.5745 |
| *Pseudomonas aestus* CMAA1215 | GCF_000474765.1 | 80.9066 | 81.5598 |
| *Pseudomonas reidholzensis* CCOS 865 | GCF_900536025.1 | 80.9176 | 81.4549 |
| *Pseudomonas aegrilactucae* MAFF 301350 | GCF_019168305.1 | 80.7954 | 81.4198 |
| *Pseudomonas chlororaphis* NBRC 3521 | GCF_000813225.1 | 81.0682 | 81.4136 |
| *Pseudomonas protegens* CHA0 | GCF_000397205.1 | 80.8627 | 81.393 |
| *Pseudomonas asiatica* RYU5 | GCF_009932335.1 | 80.9994 | 81.3691 |
| *Pseudomonas saponiphila* DSM 9751 | GCF_900105185.1 | 81.0853 | 81.3395 |
| *Pseudomonas vanderleydeniana* RW8P3 | GCF_014268755.2 | 80.8101 | 81.3249 |
| *Pseudomonas shirazica* VM14 | GCF_900291065.1 | 80.9413 | 81.312 |
| *Pseudomonas brassicae* MAFF 212427 | GCF_010671725.1 | 80.8147 | 81.2887 |
| *Pseudomonas flavescens* NBRC 103044 | GCF_002091575.1 | 80.8012 | 81.2865 |
| *Pseudomonas wadenswilerensis* CCOS 864 | GCF_900497695.1 | 80.9357 | 81.2659 |
| *Pseudomonas oryzicola* RD9SR1 | GCF_014269185.2 | 80.8916 | 81.2656 |
| *Pseudomonas phenolilytica* RBPA9 | GCF_021432765.1 | 81.1395 | 81.2335 |
| *Pseudomonas anuradhapurensis* RD8MR3 | GCF_014269225.2 | 81.0064 | 81.2268 |
| *Pseudomonas vlassakiae* RW4S2 | GCF_014269035.2 | 80.7175 | 81.2224 |
| *Pseudomonas sessilinigenes* CMR12a | GCF_003850565.1 | 80.7433 | 81.2214 |
| *Pseudomonas fluvialis* ASS-1 | GCF_002234375.1 | 80.6788 | 81.2018 |
| *Pseudomonas cuatrocienegasensis* CIP 109853 | GCF_900110925.1 | 80.9234 | 81.1886 |
| *Pseudomonas pharmacofabricae* ZYSR67-Z | GCF_002835605.1 | 80.7519 | 81.1809 |
| *Pseudomonas nitrititolerans* GL14 | GCF_003696285.1 | 80.75 | 81.1752 |
| *Pseudomonas donghuensis* HYS | GCF_000259195.1 | 80.7645 | 81.1743 |
| *Pseudomonas inefficax* JV551A3 | GCF_900277125.1 | 80.8196 | 81.167 |
| *Pseudomonas cavernicola* K1S02-6 | GCF_003596405.1 | 80.623 | 81.1439 |
| *Pseudomonas punonensis* CECT 8089 | GCF_900142655.1 | 80.6393 | 81.127 |
| *Pseudomonas capeferrum* WCS358 | GCF_000731675.1 | 80.7007 | 81.1247 |
| *Pseudomonas urethralis* BML-PP042 | GCF_013373915.1 | 80.7803 | 81.1166 |
| *Pseudomonas psychrotolerans* DSM 15758 | GCF_012985915.1 | 80.5808 | 81.0845 |
| *Pseudomonas oryzihabitans* NBRC 102199 | GCF_000730625.1 | 80.532 | 81.0551 |
| *Pseudomonas huaxiensis* WCHPs060044 | GCF_003231275.1 | 80.8162 | 81.0346 |
| *Pseudomonas putida* NBRC 14164 | GCF_000412675.1 | 80.7884 | 81.0331 |
| *Pseudomonas batumici* UCM B-321 | GCF_000820515.1 | 80.7607 | 81.0243 |
| *Pseudomonas anguilliseptica* DSM 12111 | GCF_900105355.1 | 80.7839 | 80.9412 |
| *Pseudomonas kermanshahensis* SWRI100 | GCF_014269205.2 | 80.697 | 80.9407 |
| *Stutzerimonas chloritidismutans* AW-1 | GCF_000495915.1 | 80.5612 | 80.9289 |
| *Pseudomonas kurunegalensis* RW1P2 | GCF_014269245.2 | 80.667 | 80.8985 |
| *Pseudomonas guariconensis* LMG 27394 | GCF_900102675.1 | 80.5247 | 80.874 |
| *Pseudomonas taeanensis* MS-3 | GCF_000498575.2 | 80.3285 | 80.8491 |
| *Pseudomonas songnenensis* NEAU-ST5-5 | GCF_003696315.1 | 80.7233 | 80.8294 |
| *Pseudomonas eucalypticola* NP-1 | GCF_013374995.1 | 80.4617 | 80.8172 |
| *Pseudomonas farsensis* SWRI107 | GCF_014268805.2 | 80.3661 | 80.8171 |
| *Pseudomonas peli* DSM 17833 | GCF_012986145.1 | 80.3677 | 80.8113 |
| *Pseudomonas cremoricolorata* DSM 17059 | GCF_000425745.1 | 80.5477 | 80.7881 |
| *Pseudomonas monteilii* DSM 14164 | GCF_000621245.1 | 80.5181 | 80.7484 |
| *Pseudomonas rhizoryzae* RY24 | GCF_005250615.1 | 80.4595 | 80.7474 |
| *Pseudomonas thivervalensis* DSM 13194 | GCF_001269655.1 | 80.1958 | 80.7047 |
| *Pseudomonas tructae* SNU WT1 | GCF_004214895.1 | 80.5109 | 80.6962 |
| *Pseudomonas seleniipraecipitans* LMG 25475 | GCF_900102335.1 | 80.2028 | 80.6817 |
| *Pseudomonas zarinae* SWRI108 | GCF_014268695.2 | 80.3274 | 80.6808 |
| *Pseudomonas juntendi* BML3 | GCF_009932375.1 | 80.4844 | 80.6773 |
| *Pseudomonas morbosilactucae* MAFF 302030 | GCF_023241715.1 | 80.1755 | 80.6607 |
| *Pseudomonas coleopterorum* LMG 28558 | GCF_900105555.1 | 80.1445 | 80.6328 |
| *Pseudomonas kilonensis* DSM 13647 | GCF_001269885.1 | 80.1246 | 80.591 |
| *Pseudomonas kielensis* MBT-1 | GCF_014236655.1 | 80.0506 | 80.589 |
| *Pseudomonas vranovensis* DSM 16006 | GCF_000425805.1 | 80.2219 | 80.5889 |
| *Pseudomonas urmiensis* SWRI10 | GCF_014268815.2 | 80.3795 | 80.5826 |
| *Pseudomonas brassicacearum* CCUG 51508 | GCF_008801605.1 | 80.3794 | 80.5794 |
| *Pseudomonas kribbensis* 46-2 | GCF_003352185.1 | 80.2847 | 80.561 |
| *Pseudomonas ogarae* F113 | GCF_000237065.1 | 80.2461 | 80.5504 |
| *Pseudomonas zanjanensis* SWRI12 | GCF_014268745.2 | 80.1998 | 80.5478 |
| *Pseudomonas alvandae* SWRI17 | GCF_019141525.1 | 80.109 | 80.5375 |
| *Pseudomonas taiwanensis* DSM 21245 | GCF_000425785.1 | 80.3674 | 80.5225 |
| *Pseudomonas ceruminis* BML-PP028 | GCF_013373895.1 | 80.2878 | 80.5203 |
| *Pseudomonas aylmerensis* S1E40 | GCF_001702265.1 | 80.0825 | 80.509 |
| *Pseudomonas alkylphenolica* KL28 | GCF_000746525.1 | 80.3024 | 80.5053 |
| *Pseudomonas fuscovaginae* LMG 2158 | GCF_900108595.1 | 80.2683 | 80.5047 |
| *Pseudomonas asplenii* ATCC 23835 | GCF_900105475.1 | 80.356 | 80.4943 |
| *Pseudomonas bijieensis* L22-9 | GCF_013347965.1 | 80.2333 | 80.4714 |
| *Pseudomonas veronii* DSM 11331 | GCF_001439695.1 | 80.1498 | 80.4569 |
| *Pseudomonas saudiphocaensis* 20_BN | GCF_000756775.1 | 80.1035 | 80.4519 |
| *Pseudomonas canavaninivorans* HB002 | GCF_016405165.1 | 79.9636 | 80.4323 |
| *Pseudomonas mediterranea* CFBP 5447 | GCF_000774145.1 | 80.1775 | 80.4317 |
| *Pseudomonas trivialis* DSM 14937 | GCF_001439805.1 | 80.011 | 80.4146 |
| *Pseudomonas ovata* F51 | GCF_003131185.1 | 80.0487 | 80.4041 |
| *Pseudomonas azotoformans* LMG 21611 | GCF_001870415.1 | 79.9735 | 80.3996 |
| *Pseudomonas baltica* MBT-2 | GCF_014235765.1 | 80.011 | 80.3995 |
| *Pseudomonas botevensis* COW3 | GCF_019145475.1 | 80.1112 | 80.3866 |
| *Pseudomonas extremaustralis* 14-3 substr. 14-3b | GCF_000242115.1 | 79.9743 | 80.3816 |
| *Pseudomonas allii* MAFF 301514 | GCF_013392005.1 | 80.0704 | 80.3584 |
| *Pseudomonas shirazensis* SWRI56 | GCF_014268785.2 | 79.8927 | 80.3565 |
| *Pseudomonas pergaminensis* 1008 | GCF_024112395.1 | 79.7714 | 80.3558 |
| *Pseudomonas gozinkensis* IzPS32d | GCF_014863585.1 | 80.0921 | 80.3468 |
| *Pseudomonas parafulva* DSM 17004 | GCF_000425765.1 | 80.0766 | 80.3465 |
| *Pseudomonas viciae* 11K1 | GCF_004786035.1 | 79.9821 | 80.3351 |
| *Pseudomonas fitomaticsae* FIT81 | GCF_021018765.1 | 80.1473 | 80.3244 |
| *Pseudomonas pharyngis* BML-PP036 | GCF_021602345.1 | 80.0696 | 80.32 |
| *Pseudomonas azerbaijanoriens* SWRI123 | GCF_019139795.1 | 79.9009 | 80.3066 |
| *Pseudomonas allokribbensis* IzPS23 | GCF_014863605.1 | 80.0622 | 80.2843 |
| *Pseudomonas glycinae* MS586 | GCF_001594225.2 | 80.0877 | 80.2812 |
| *Pseudomonas rhizosphaerae* DSM 16299 | GCF_000761155.1 | 80.2408 | 80.28 |
| *Pseudomonas paraglycinae* BML-PP023 | GCF_021601625.1 | 80.0405 | 80.2793 |
| *Pseudomonas extremorientalis* LMG 19695 | GCF_001870465.1 | 79.8252 | 80.2739 |
| *Pseudomonas sputi* BML-PP014 | GCF_021603585.1 | 79.987 | 80.2214 |
| *Pseudomonas parakoreensis* BML-PP030 | GCF_021602155.1 | 79.6338 | 80.2177 |
| *Pseudomonas salmasensis* SWRI126 | GCF_014268375.2 | 79.8281 | 80.2084 |
| *Pseudomonas cedrina* DSM 17516 | GCF_001983175.1 | 79.8644 | 80.208 |
| *Serpens gallinarum* Sa2CUA2 | GCF_014836765.1 | 79.9481 | 80.2074 |
| *Pseudomonas shahriarae* SWRI52 | GCF_014268455.2 | 79.923 | 80.2034 |
| *Pseudomonas bananamidigenes* BW11P2 | GCF_001679645.1 | 79.8647 | 80.2012 |
| *Pseudomonas koreensis* CCUG 51519 | GCF_008801535.1 | 79.8319 | 80.1864 |
| *Pseudomonas vancouverensis* Dha-51 | GCF_004348895.1 | 79.762 | 80.1836 |
| *Pseudomonas triticicola* SWRI88 | GCF_019145375.1 | 79.8556 | 80.183 |
| *Pseudomonas marvdashtae* SWRI102 | GCF_014268655.2 | 79.9009 | 80.1796 |
| *Pseudomonas gessardii* DSM 17152 | GCF_001983165.1 | 79.9092 | 80.1705 |
| *Pseudomonas jessenii* DSM 17150 | GCF_002236115.1 | 79.7559 | 80.1609 |
| *Pseudomonas akapageensis* PS24 | GCF_011355085.1 | 80.1098 | 80.1544 |
| *Pseudomonas azadiae* SWRI103 | GCF_019145355.1 | 79.82 | 80.1542 |
| *Pseudomonas laurylsulfatiphila* AP3_16 | GCF_002934665.1 | 79.8019 | 80.1513 |
| *Pseudomonas simiae* CCUG 50988 | GCF_001730615.1 | 79.7908 | 80.1437 |
| *Pseudomonas siliginis* SWRI31 | GCF_019145195.1 | 79.7526 | 80.137 |
| *Pseudomonas poae* DSM 14936 | GCF_001439785.1 | 79.8737 | 80.1286 |
| *Pseudomonas iranensis* SWRI54 | GCF_014268585.2 | 79.725 | 80.1079 |
| *Pseudomonas tolaasii* NCPPB 2192 | GCF_002813445.1 | 79.7222 | 80.1045 |
| *Pseudomonas promysalinigenes* RW10S1 | GCF_014269025.2 | 79.7386 | 80.103 |
| *Pseudomonas uvaldensis* 20TX0172 | GCF_021271205.1 | 79.7537 | 80.0975 |
| *Pseudomonas lurida* LMG 21995 | GCF_002563895.1 | 79.9025 | 80.0799 |
| *Pseudomonas cyclaminis* MAFF 301449 | GCF_015163715.1 | 79.795 | 80.0713 |
| *Pseudomonas yamanorum* LMG 27247 | GCF_900105735.1 | 79.8511 | 80.0694 |
| *Pseudomonas corrugata* DSM 7228 | GCF_001269905.1 | 79.8791 | 80.0625 |
| *Pseudomonas proteolytica* DSM 15321 | GCF_007858275.1 | 79.7033 | 80.0519 |
| *Pseudomonas canadensis* Feb-92 | GCF_000503215.1 | 79.884 | 80.0355 |
| *Pseudomonas lactucae* MAFF 301380 | GCF_016937615.1 | 79.8482 | 79.9995 |
| *Pseudomonas grimontii* DSM 17515 | GCF_007858185.1 | 79.8136 | 79.9949 |
| *Pseudomonas tehranensis* SWRI196 | GCF_014268615.1 | 79.7973 | 79.9897 |
| *Pseudomonas umsongensis* DSM 16611 | GCF_002236105.1 | 79.6876 | 79.9848 |
| *Pseudomonas brenneri* DSM 15294 | GCF_007858285.1 | 79.666 | 79.9818 |
| *Pseudomonas izuensis* IzPS43_3003 | GCF_009861505.1 | 79.5754 | 79.9725 |
| *Pseudomonas atacamensis* M7D1 | GCF_004801935.1 | 79.7941 | 79.9724 |
| *Pseudomonas petroselini* MAFF 311094 | GCF_021166635.1 | 79.7138 | 79.9554 |
| *Pseudomonas lactis* DSM 29167 | GCF_001439845.1 | 79.7986 | 79.951 |
| *Pseudomonas orientalis* DSM 17489 | GCF_001439815.1 | 79.7663 | 79.948 |
| *Pseudomonas asgharzadehiana* SWRI132 | GCF_019139815.1 | 79.7854 | 79.9435 |
| *Pseudomonas fildesensis* KG01 | GCF_001050345.1 | 79.6019 | 79.9413 |
| *Pseudomonas paracarnis* V5/DAB/2/5 | GCF_904063055.1 | 79.811 | 79.9344 |
| *Pseudomonas monsensis* PGSB 8459 | GCF_014268495.2 | 79.7835 | 79.9338 |
| *Pseudomonas hamedanensis* SWRI65 | GCF_014268595.2 | 79.5837 | 79.9303 |
| *Pseudomonas fulva* DSM 17717 | GCF_000621265.1 | 79.7047 | 79.9261 |
| *Pseudomonas marginalis* ICMP 3553 | GCF_001645105.1 | 79.7571 | 79.9196 |
| *Pseudomonas khavaziana* SWRI124 | GCF_019145205.1 | 79.528 | 79.8995 |
| *Pseudomonas libanensis* DSM 17149 | GCF_001439685.1 | 79.6799 | 79.8947 |
| *Pseudomonas synxantha* DSM 18928 | GCF_001439725.1 | 79.7032 | 79.8827 |
| *Pseudomonas nabeulensis* E10B | GCF_004682045.1 | 79.6134 | 79.8757 |
| *Pseudomonas rhizophila* S211 | GCF_003033885.1 | 79.8547 | 79.8747 |
| *Pseudomonas defluvii* WCHP16 | GCF_001695625.1 | 79.6593 | 79.8706 |
| *Pseudomonas salomonii* LMG 22120 | GCF_001730645.1 | 79.6903 | 79.8633 |
| *Pseudomonas rhodesiae* DSM 14020 | GCF_007858255.1 | 79.6532 | 79.8614 |
| *Pseudomonas gregormendelii* LMG 28632 | GCF_017114825.1 | 79.573 | 79.8589 |
| *Pseudomonas pisciculturae* P115 | GCF_015461805.1 | 79.473 | 79.8583 |
| *Pseudomonas mohnii* DSM 18327 | GCF_900105115.1 | 79.6497 | 79.8583 |
| *Pseudomonas laurylsulfativorans* AP3_22 | GCF_002906155.1 | 79.6235 | 79.8488 |
| *Pseudomonas baetica* LMG 25716 | GCF_002813455.1 | 79.3646 | 79.8475 |
| *Pseudomonas leptonychotis* CCM 8849 | GCF_004920405.1 | 79.5926 | 79.8366 |
| *Pseudomonas kairouanensis* KC12 | GCF_004682055.1 | 79.689 | 79.8359 |
| *Pseudomonas silesiensis* A3 | GCF_001661075.1 | 79.7868 | 79.8267 |
| *Pseudomonas paralactis* DSM 29164 | GCF_001439735.1 | 79.6501 | 79.8256 |
| *Pseudomonas tritici* SWRI145 | GCF_014268275.3 | 79.7217 | 79.823 |
| *Pseudomonas haemolytica* DSM 108987 | GCF_009659625.1 | 79.6773 | 79.8129 |
| *Pseudomonas massiliensis* CB1 | GCF_000826105.1 | 79.4 | 79.7917 |
| *Pseudomonas atagonensis* PS14 | GCF_011369485.1 | 79.4488 | 79.7614 |
| *Pseudomonas palleroniana* LMG 23076 | GCF_003031675.1 | 79.4811 | 79.7573 |
| *Pseudomonas laurentiana* JCM 32154 | GCF_014648275.1 | 79.3971 | 79.7551 |
| *Pseudomonas granadensis* LMG 27940 | GCF_900105485.1 | 79.6286 | 79.7514 |
| *Pseudomonas zeae* OE 48.2 | GCF_014268485.2 | 79.4459 | 79.7225 |
| *Pseudomonas khorasanensis* SWRI153 | GCF_014268505.2 | 79.3449 | 79.7065 |
| *Pseudomonas moorei* CCUG 53114 | GCF_008801475.1 | 79.6354 | 79.7033 |
| *Pseudomonas fluorescens* DSM 50090 | GCF_001269845.1 | 79.4593 | 79.7021 |
| *Pseudomonas migulae* NBRC 103157 | GCF_002091715.1 | 79.5156 | 79.7011 |
| *Pseudomonas arsenicoxydans* CECT 7543 | GCF_900103875.1 | 79.3152 | 79.6897 |
| *Pseudomonas sivasensis* P7 | GCF_013778505.1 | 79.4303 | 79.6878 |
| *Pseudomonas reinekei* MT1 | GCF_001945365.1 | 79.4482 | 79.6861 |
| *Pseudomonas agarici* NCPPB 2289 | GCF_000280785.1 | 79.3951 | 79.6403 |
| *Pseudomonas edaphica* RD25 | GCF_005863185.1 | 79.4551 | 79.6158 |
| *Pseudomonas antarctica* CMS 35 | GCF_010634845.1 | 79.3212 | 79.6057 |
| *Pseudomonas cremoris* WS 5106 | GCF_014230465.1 | 79.7002 | 79.6048 |
| *Pseudomonas costantinii* LMG 22119 | GCF_001870435.1 | 79.4712 | 79.5997 |
| *Pseudomonas tensinigenes* ZA 5.3 | GCF_014268445.2 | 79.4301 | 79.5972 |
| *Pseudomonas azerbaijanoccidens* SWRI74 | GCF_019145495.1 | 79.5347 | 79.5881 |
| *Pseudomonas crudilactis* UCMA 17988 | GCF_013433315.1 | 79.1886 | 79.5654 |
| *Pseudomonas mucoides* P154a | GCF_015461845.1 | 79.4165 | 79.5362 |
| *Pseudomonas germanica* FIT28 | GCF_019614655.1 | 79.3174 | 79.5344 |
| *Pseudomonas neuropathica* P155 | GCF_015461835.1 | 79.3882 | 79.5336 |
| *Pseudomonas farris* SWRI79 | GCF_019145235.1 | 79.3008 | 79.5229 |
| *Pseudomonas spelaei* CCM 7893 | GCF_009724245.1 | 79.5416 | 79.5006 |
| *Pseudomonas guineae* LMG 24016 | GCF_900113745.1 | 79.3058 | 79.4874 |
| *Pseudomonas rustica* MBT-4 | GCF_018336155.1 | 79.2476 | 79.4824 |
| *Pseudomonas lini* DSM 16768 | GCF_001042905.1 | 79.3498 | 79.4744 |
| *Pseudomonas prosekii* LMG 26867 | GCF_900105155.1 | 79.2148 | 79.4693 |
| *Pseudomonas mandelii* NBRC 103147 | GCF_002091695.1 | 79.326 | 79.4634 |
| *Pseudomonas kitaguniensis* MAFF 212408 | GCF_009296165.1 | 79.223 | 79.4414 |
| *Pseudomonas pohangensis* DSM 17875 | GCF_900105995.1 | 79.6228 | 79.4394 |
| *Pseudomonas karstica* CCM 7891 | GCF_009707515.1 | 79.1 | 79.2837 |
| *Pseudomonas viridiflava* DSM 6694 | GCF_001305955.1 | 79.2583 | 79.2578 |
| *Pseudomonas syringae* KCTC 12500 | GCF_000507185.2 | 79.3216 | 79.2574 |
| *Pseudomonas mucidolens* NBRC 103159 | GCF_002091735.1 | 79.1691 | 79.2519 |
| *Pseudomonas capsici* Pc19-1 | GCF_017165765.1 | 79.0906 | 79.2323 |
| *Pseudomonas floridensis* GEV388 | GCF_002087235.1 | 79.0372 | 79.1972 |
| *Pseudomonas saudimassiliensis* 12M76_air | GCF_000939975.1 | 79.1216 | 79.1839 |
| *Pseudomonas fragi* NBRC 3458 | GCF_002091615.1 | 79.2529 | 79.1777 |
| *Pseudomonas bohemica* IA19 | GCF_002934685.1 | 79.2766 | 79.1475 |
| *Pseudomonas graminis* DSM 11363 | GCF_900111735.1 | 78.9767 | 79.1461 |
| *Pseudomonas abietaniphila* ATCC 700689 | GCF_900100795.1 | 79.0925 | 79.1382 |
| *Pseudomonas arcuscaelestis* P66 | GCF_016881005.1 | 78.9368 | 79.1253 |
| *Pseudomonas avellanae* BPIC 631 | GCF_000302915.1 | 79.0289 | 79.1068 |
| *Pseudomonas triticumensis* DOAB 1067 | GCF_014358015.1 | 79.1648 | 79.0927 |
| *Pseudomonas congelans* DSM 14939 | GCF_900103225.1 | 78.9848 | 79.0904 |
| *Pseudomonas asturiensis* LMG 26898 | GCF_900143095.1 | 79.1248 | 79.0857 |
| *Pseudomonas alliivorans* 20GA0068 | GCF_017826695.1 | 78.9474 | 79.0629 |
| *Pseudomonas meliae* CFBP 3225 | GCF_000935675.1 | 78.7561 | 79.0388 |
| *Pseudomonas cichorii* LMG 2162 | GCF_015471425.1 | 79.0686 | 79.0246 |
| *Pseudomonas cannabina* ICMP 2823 | GCF_001400175.1 | 79.0087 | 78.9966 |
| *Pseudomonas amygdali* CFBP 3205 | GCF_000935645.1 | 78.8734 | 78.9889 |
| *Pseudomonas ficuserectae* ICMP 7848 | GCF_001400815.1 | 78.635 | 78.897 |
| *Pseudomonas typographi* CA3A | GCF_014694385.1 | 78.7089 | 78.8926 |
| *Pseudomonas savastanoi* ICMP 4352 | GCF_001401285.1 | 78.7931 | 78.8715 |
| *Pseudomonas caricapapayae* ICMP 2855 | GCF_001400735.1 | 78.7634 | 78.8702 |
| *Pseudomonas versuta* L10.10 | GCF_001294575.1 | 78.6726 | 78.8262 |
| *Pseudomonas paraversuta* V4/DAB/S4/2a | GCF_904063065.1 | 78.7622 | 78.8243 |
| *Pseudomonas deceptionensis* DSM 26521 | GCF_001042895.1 | 78.5365 | 78.8191 |
| *Pseudomonas segetis CIP* 108523 | GCF_900188155.1 | 78.4775 | 78.6898 |
| *Pseudomonas lundensis* DSM 6252 | GCF_001042985.1 | 78.4748 | 78.6793 |
| *Pseudomonas marincola* JCM 14761 | GCF_900116605.1 | 78.4317 | 78.6526 |
| *Pseudomonas tremae* ICMP 9151 | GCF_001401155.1 | 78.6736 | 78.6497 |
| *Pseudomonas matsuisoli* JCM 30078 | GCF_014647635.1 | 78.7151 | 78.6289 |
| *Pseudomonas quercus* hsmgli-8 | GCF_012033695.1 | 78.4094 | 78.6213 |
| *Pseudomonas psychrophila* DSM 17535 | GCF_001043005.1 | 78.4859 | 78.6177 |
| *Pseudomonas abyssi* MT5 | GCF_002307495.1 | 78.658 | 78.5948 |
| *Pseudomonas phragmitis* S-6-2 | GCF_002056295.1 | 78.4766 | 78.5859 |
| *Pseudomonas helleri* DSM 29165 | GCF_001043025.1 | 78.838 | 78.5746 |
| *Pseudomonas foliumensis* DOAB 1069 | GCF_014357575.1 | 78.2522 | 78.5744 |
| *Pseudomonas yangmingensis* DSM 24213 | GCF_900114825.1 | 78.7831 | 78.564 |
| *Pseudomonas caspiana* FBF102 | GCF_002158995.1 | 78.4263 | 78.5505 |
| *Pseudomonas californiensis* CDFA601 | GCF_021147775.1 | 78.6492 | 78.5433 |
| *Pseudomonas quasicaspiana* CDFA553 | GCF_021147825.1 | 78.2288 | 78.5076 |
| *Pseudomonas weihenstephanensis* DSM 29166 | GCF_001043055.1 | 78.3869 | 78.461 |
| *Pseudomonas taetrolens* DSM 21104 | GCF_001042915.1 | 78.4667 | 78.455 |
| *Pseudomonas nanhaiensis* SCS 2-3 | GCF_020025155.1 | 78.0418 | 78.4197 |
| *Pseudomonas jilinensis JS15-10A1* | GCF_003586265.1 | 78.1687 | 78.408 |
| *Pseudomonas saxonica* DSM 108989 | GCF_007858365.1 | 77.8713 | 78.212 |
| *Pseudomonas endophytica* BSTT44 | GCF_001411475.1 | - | 77.8417 |
| *Pseudomonas laoshanensis* Y22 | GCF_008365385.1 | - | 77.4018 |

**Table S3.** The sensitivity of strains Q1-7 and Q4-3 to different antibiotics

| Antibiotics | Minimal inhibitory concentration (μg/mL) | |
| --- | --- | --- |
|  | Q1-7 | Q4-3 |
| Ampicillin | >40 | >640 |
| Kanamycin | >5 | >10 |
| Streptomycin | >10 | >40 |
| Gentamicin | >5 | >5 |
| Tetracycline | >5 | >5 |
| Polymyxin B | >5 | >5 |

**Table S4.** Primers used in this study

| / | Primer | Primer sequence (5’-3’) | Gene segment |
| --- | --- | --- | --- |
| Reference gene | infB-F | TGGTCTCTTCCTTCCTCTC | *infB* |
|  | infB-F | CGTCATCATCGGCTTCAA |  |
| RT-PCR primers | phcA-F | CGAATGCGAGCATATCTTC | *phcA* |
|  | phcA-R | TCCTTCATCAGCGAGTTG |  |
|  | phcB-F | CGTCTATCGCACCTACAC | *phcB* |
|  | phcB-R | CGAGATAGTTGACCAGGATAT |  |
|  | phcS-F | CGAACGACTGCATGATCC | *phcS* |
|  | phcS-R | ATCCGCATCGAAGACAAC |  |
|  | solI-F | CGCTATCGCTACAAGGTAT | *solI* |
|  | solI-R | AGACGCTTTCCAGCAAAT |  |
|  | solR-F | CTCATCACGGATGGACAC | *solR* |
|  | solR-R | TTGACTACTGCTGCTACG |  |
|  | epsB-F | CGCTGGAAGGCAAGAATC | *epsB* |
|  | epsB-R | CGCATCTCGTTGTACTTCA |  |
|  | cbhA-F | GTCTGGCTTGATAGTATTGG | *cbhA* |
|  | cbhA-R | ACCGTAATTGGCTTATTCG |  |
|  | endoglunase-F | GCGAAGGCGTTGTATGAA | *endoglunase* |
|  | endoglunase-F | CGATCCGATCACCAAGGA |  |
|  | pme-F | TGTTCTTGAAGGTCAGGTT | *pme* |
|  | pme-F | TCTACAACGAACTGGTCTG |  |

**Table S5.** Comparison of reported esterase protein sequences based on the protein sequences of Q1-7 and Q4-3

| Known protein | Q1-7 | | | Q4-3 | | |
| --- | --- | --- | --- | --- | --- | --- |
|  | Gene locus | Identity (coverage) % | Function | Gene locus | Identity (coverage)% | Function |
| βHPMEH (AB204804.1) | tmp_000251 | 8.61 (29.20) | hydrolase | GE003338 | 3.78 (13.66) | Diguanylate cyclase DgcP |
|  |  |  |  | GE002153 | 7.98 (26.05) | Acetyl-hydrolase |
| ELP86 (MF279069) | tmp_001873 | 28.42 (88.49) | alpha/beta hydrolase | GE001437 | 29.13 (91.01) | Putative aminoacrylate hydrolase RutD |
|  | tmp_000650 | 24.10 (76.98) | alpha/beta hydrolase | GE002701 | 30.94 (91.37) | 3-oxoadipate enol-lactonase |
|  | tmp_004909 | 27.34 (87.05) | poly(3-hydroxyalkanoate) depolymerase | GE005428 | 27.34 (89.92) | Poly(3-hydroxyalkanoate) depolymerase |
|  | tmp_002772 | 24.82 (80.58) | oxoadipate enol-lactonase | GE002832 | 26.98 (83.45) | 3-oxoadipate enol-lactonase 2 |
|  | tmp_001540 | 30.94 (87.05) | alpha/beta fold hydrolase | GE004609 | 31.65 (96.76) | Rhodomycin D methylesterase |
|  | tmp_003517 | 25.18 (92.81) | alpha/beta hydrolase | GE001762 | 26.26 (96.42) | Lipase 1 |
|  | tmp_000885 | 23.74 (97.48) | alpha/beta hydrolase | GE003321 | 23.74 (92.09) | Arylesterase |
|  | tmp_001184 | 26.61 (91.37) | alpha/beta hydrolase | GE002031 | 30.22 (95.32) | Epoxide hydrolase A |
|  | tmp_004031 | 22.66 (89.21) | alpha/beta hydrolase | GE004690 | 28.05 (95.68) | 2-hydroxy-6- oxononatrienedioate hydrolase |
|  |  |  |  | GE002312 | 24.82 (93.17) | Dihydrolipoyllysine-residue acetyltransferase component of acetoin cleaving system |
|  |  |  |  | GE005792 | 24.46 (89.93) | hypothetical protein |
|  |  |  |  | GE002299 | 23.02 (93.53) | Non-heme chloroperoxidase |
|  |  |  |  | GE004915 | 21.94 (87.41) | 2-hydroxy-6- oxononatrienedioate hydrolase |
|  |  |  |  | GE002456 | 23.74 (86.33) | 4,5:9,10-diseco-3-hydroxy-5,9, 17-trioxoandrosta-1(10),2-diene-4-oate hydrolase |
| ELP96 (MF279070) | tmp_002349 | 4.83 (10.88) | alpha/beta hydrolase | GE005408 | 17.52 (67.37) | Chain Glutamate synthase large |
| ELP104 (MF279071) | tmp_003517 | 9.02 (28.09) | alpha/beta hydrolase | GE001572 | 10.82 (36.34) | hypothetical protein |
|  | tmp_003849 | 9.28 (34.54) | alpha/beta fold hydrolase | GE005792 | 10.82 (25.51) | hypothetical protein |
|  | tmp_000650 | 9.28 (33.25) | alpha/beta hydrolase | GE001762 | 10.82 (40.72) | Lipase 1 |
| EstDL33 (MF279068) | tmp_002686 | 18.02 (67.26) | beta-lactamase | GE001894 | 22.33 (83.24) | Beta-lactamase |
|  | tmp_001744 | 19.05 (51.52) | beta-lactamase family protein | GE001366 | 15.23 (56.85) | D-alanyl-D-alanine carboxypeptidase |
|  | tmp_001341 | 13.45 (53.30) | beta-lactamase family protein | GE001260 | 10.15 (40.86) | hypothetical protein |
